# Risk-stratified monitoring for thiopurine toxicity in immune-mediated inflammatory diseases: prognostic model development, validation, and, health economic evaluation

**DOI:** 10.1016/j.eclinm.2023.102213

**Published:** 2023-09-14

**Authors:** Georgina Nakafero, Tim Card, Matthew J. Grainge, Hywel C. Williams, Maarten W. Taal, Guruprasad P. Aithal, Christopher P. Fox, Christian D. Mallen, Danielle A. van der Windt, Matthew D. Stevenson, Richard D. Riley, Abhishek Abhishek

**Affiliations:** a Academic Rheumatology, School of Medicine, University of Nottingham, Nottingham NG5 1PB, UK; b Lifespan and Population Health, School of Medicine, University of Nottingham, Nottingham NG5 1PB, UK; c Centre for Kidney Research and Innovation, School of Medicine, Translational Medical Sciences, University of Nottingham, Derby DE22 3NE, UK; d Nottingham Digestive Diseases Centre, Translational Medical Sciences, School of Medicine, University of Nottingham, Nottingham NG7 2UH, UK; e Translational Medical Sciences, School of Medicine, University of Nottingham, Nottingham, UK; f Primary Care Centre Versus Arthritis, School of Medicine, Keele University, Keele ST5 5BJ, UK; g School of Health and Related Research, University of Sheffield, Sheffield S1 4DA, UK; h Institute of Applied Health Research, College of Medical and Dental Sciences, University of Birmingham, Birmingham B15 2TT, UK

**Keywords:** Inflammatory bowel disease, Thiopurine, Drug toxicity prediction

## Abstract

**Background:**

Patients established on thiopurines (e.g., azathioprine) are recommended to undergo three-monthly blood tests for the early detection of blood, liver, or kidney toxicity. These side-effects are uncommon during long-term treatment. We developed a prognostic model that could be used to inform risk-stratified decisions on frequency of monitoring blood-tests during long-term thiopurine treatment, and, performed health-economic evaluation of alternate monitoring intervals.

**Methods:**

This was a retrospective cohort study set in the UK primary-care. Data from the Clinical Practice Research Datalink Aurum and Gold formed development and validation cohorts, respectively. People age ≥18 years, diagnosed with an immune mediated inflammatory disease, prescribed thiopurine by their general practitioner for at-least six-months between January 1, 2007 and December 31, 2019 were eligible. The outcome was thiopurine discontinuation with abnormal blood-test results. Patients were followed up from six-months after first primary-care thiopurine prescription to up to five-years. Penalised Cox regression developed the risk equation. Multiple imputation handled missing predictor data. Calibration and discrimination assessed model performance. A mathematical model evaluated costs and quality-adjusted life years associated with lengthening the interval between blood-tests.

**Findings:**

Data from 5982 (405 events over 16,117 person-years) and 3573 (269 events over 9075 person-years) participants were included in the development and validation cohorts, respectively. Fourteen candidate predictors (21 parameters) were included. The optimism adjusted R^2^ and Royston D statistic in development data were 0.11 and 0.76, respectively. The calibration slope and Royston D statistic (95% Confidence Interval) in the validation data were 1.10 (0.84–1.36) and 0.72 (0.52–0.92), respectively. A 2-year period between monitoring blood-test was most cost-effective in all deciles of predicted risk but the gain between monitoring annually or biennially reduced in higher risk deciles.

**Interpretation:**

This prognostic model requires information that is readily available during routine clinical care and may be used to risk-stratify blood-test monitoring for thiopurine toxicity. These findings should be considered by specialist societies when recommending blood monitoring during thiopurine prescription to bring about sustainable and equitable change in clinical practice.

**Funding:**

10.13039/501100000272National Institute for Health and Care Research.

**Research in context**

**Evidence before this study**

Patients established on a thiopurine (e.g., azathioprine) are recommended to undergo three-monthly monitoring blood tests for the early detection of blood, liver, or kidney toxicity even though these side-effects are uncommon during long-term treatment. Our review of research indexed in the PubMed between 1st January 1995 and 31st December 2022 using search terms: thiopurine AND monitoring AND cost-effective without any restrictions on language and study-design did not find any study that evaluated the effectiveness and/or cost-effectiveness of blood-test monitoring stratified according to the individuals’ risk of toxicity during long-term thiopurine treatment.

**Added value of this study**

In this study we set out to find whether the risk of clinically significant liver, blood, or kidney toxicity during established thiopurine treatment can be predicted and the interval between monitoring blood-tests be increased cost-effectively. We found that in adults with a broad range of immune mediated inflammatory diseases, a prognostic model that included demographic and clinical features that are easily ascertained during clinic visits, predicted clinically-significant blood-test abnormalities with high calibration and discrimination. The model performance was comparable across age groups and in people with inflammatory bowel disease. It was also cost-effective to increase the interval between monitoring blood-tests.

**Implications of all the available evidence**

Patients and health professionals may decide the interval between monitoring blood-tests during long-term thiopurine treatment using this easy-to-use prediction model. Health policy makers may use the risk scores alongside the cost-effectiveness estimates to recommend the risk threshold at which the intervals between monitoring blood-tests may be increased. Future research is required to ascertain whether the addition of results of thiopurine methyl transferase testing and thiopurine therapeutic drug monitoring metabolites to the model would improve its performance.

## Introduction

Thiopurines are a cornerstone glucocorticoid sparing drug for the treatment of inflammatory bowel diseases (IBD), widely recommended globally for maintenance of remission in ulcerative colitis (UC) and Crohn’s disease (CD).^1^, ^2^, ^3^, ^4^ They are among first-line glucocorticoid sparing drugs for the treatment of systemic lupus erythematosus (SLE), and are used in the management of rheumatoid arthritis (RA) and psoriasis ± arthritis.^5^^,^^6^ The usual recommended dose for the management of IBD and other inflammatory conditions is azathioprine 2.0–2.5 mg/kg/day, and 1.0–3.0 mg/kg/day, respectively, although lower doses may be effective.

Thiopurines can cause myelotoxicity, hepatotoxicity, and, nephrotoxicity, mostly during the first few months of treatment, and dose-reduction is considered in severe renal impairment.^7^, ^8^, ^9^, ^10^, ^11^, ^12^ Concern about these issues led to recommendations to undertake two to four weekly monitoring blood-tests during the first few months of treatment followed by testing at three monthly intervals thereafter.^4^^,^^6^^,^^13^ Frequent monitoring blood-tests within the first few months of treatment are important due to the high risk of reversible target organ damage in this period. However, regular monitoring blood-tests during established long-term thiopurine treatment, a period when blood-test abnormalities are uncommon, seems unduly cautious and unnecessary. Whether routinely available clinical data can be used to ascertain those at low, medium, or high risk of toxicity in this period, and be used to inform risk-stratified monitoring has not been evaluated.

Avoidable testing is a wasteful use of healthcare resources. It would be beneficial to predict those at high risk of toxicity during long-term thiopurine treatment who continue to need monitoring at the current frequency while others have less frequent monitoring blood-tests. In order to inform such risk-stratified monitoring, we developed and validated a prognostic model for clinically-significant thiopurine toxicity detectable on monitoring blood-tests. We also undertook health economic analysis to evaluate the cost-effectiveness of different monitoring intervals.

## Methods

### Study setting

Data from the Clinical Practice Research Datalink (CPRD) Aurum and Gold were used for model development and validation, respectively.^14^^,^^15^ CPRD is an anonymised longitudinal database of electronic health records from primary care in the National Health Service. This study was approved by CPRD’s Research Data Governance (protocol: 20_000236R), which has overarching research ethics committee approval for studies using anonymous data (reference 05/MRE04/87). Practices that contributed data to the CPRD consented to using anonymized patient data for approved research projects and additional consent was not required prior to individual studies.

### Study design and participants

This was a retrospective cohort study. Adults (age ≥18 years), newly diagnosed with either IBD, SLE, RA, psoriasis ± arthritis, or axial spondyloarthropathy between 01/01/2007 and 12/31/2019 and prescribed thiopurine (either azathioprine or mercaptopurine) by their general-practitioner for at-least six months were included. Additionally, they were required to have ≥1-year inflammatory disease-free registration at their general-practice to be considered as being newly diagnosed,^16^^,^^17^ and to receive their first thiopurine prescription either after this diagnosis or within the preceding 90-days to allow for recording of diagnoses lagging behind prescriptions. We did not include thioguanine in this study as it is rarely used to treat inflammatory conditions. Its use is restricted to specialist centres for the treatment of refractory IBD and it is not prescribed from primary care. In the UK, it is licenced for the treatment of acute or chronic myeloid leukaemia.

Patients with either severe chronic liver disease, chronic kidney disease (CKD) stage 4 or 5, or severe haematological disease prior to start of follow-up were excluded as the former two are relative contraindications for thiopurine and inclusion of severe haematological diseases would have caused uncertainty in outcome ascertainment. Any general-practices that contributed data to both CPRD Aurum and Gold were excluded from the development cohort using a bridging file provided by the CPRD.

In the UK, thiopurine initiation and initial monitoring are led by hospital out-patient clinics. Once disease control is achieved with a stable thiopurine dose, usually four to six months after treatment initiation, the responsibility for prescribing and monitoring is handed to the patients’ general practitioner (GP).^18^, ^19^, ^20^ During shared-care prescribing all treatment changes including for abnormal blood-test results are directed by the hospital specialist.

We followed-up patients from 180-days after the first primary-care thiopurine prescription until the earliest date of outcome, death, transfer out of practice, 90-day prescription gap, last data collection from practice, 31/12/2019 or five-years. Thiopurine-toxicity associated drug discontinuation defined as a prescription gap of ≥90 days with either an abnormal blood-test result or a diagnostic code within ±60 days of the last prescription date was the outcome of interest.^21^ The threshold for abnormal blood-test results were: total leucocyte count <3.5 × 10^9^/l; neutrophil count <1.6 × 10^9^/l; platelet count <140 × 10^9^/l; ALT/AST >100 IU/ml; and kidney function decline defined as either CKD progression based on medical codes recorded by the GP, or a creatinine increase of >26 μmol/l, the threshold for consideration of acute kidney injury.^18^^,^^22^

Predictors were defined based on the latest record within 2 years of the start of follow-up except for prescriptions which were defined using the prior 6-months’ primary-care prescriptions. These were selected by clinical members of the team (Table 1). Age, sex, body mass index (BMI), alcohol intake, and, diabetes were included as they associate with drug induced liver injury.^23^ Smoking was included as it associates with active RA, CD, and, SLE that may require the use of higher thiopurine doses.^24^, ^25^, ^26^ CKD was included as it reduces thiopurine clearance.^27^ The mercaptopurine equivalent dose was included in the model as thiopurine toxicity is dose dependent.^28^^,^^29^ Statins and ACE inhibitors were included as their use is associated with target organ thiopurine toxicity.^12^ Allopurinol was included as it inhibits thiopurine metabolism by inhibiting xanthine oxidase and co-prescription can cause cytopenia if the dose of thiopurine is not reduced substantially e.g., to 25% of the regular dose.^12^ Additionally, low-dose allopurinol may be combined with low-dose azathioprine in patients with prior thiopurine induced hepatotoxicity to minimise the future risk of hepatotoxicity and myelotoxicity in hyper-methylators and this approach was shown to have greater likelihood of achieving remission in a clinical trial.^30^, ^31^, ^32^, ^33^, ^34^ Sulfasalazine, 5-aminosalicylates (5-ASA) and other immune-suppressing drugs were included as they can cause cytopenia, elevated liver enzymes, and, acute kidney injury. Additionally, 5-ASA drugs are associated with thiopurine induced myelotoxicity as they increase 6-thioguanine nucleotide (6-TGN) levels.^35^^,^^36^ Either cytopenia (neutrophil count <2 × 10^9^/l, total leucocyte count <4 × 10^9^/l, or platelet count <150 × 10^9^/l) or elevated transaminase (ALT and/or AST >35 IU/l) during the first six months of primary-care prescription were included as they predicted cytopenia and/or transaminitis in other studies.^37^^,^^38^

**Table 1:** Distribution of candidate predictors in development and validation cohorts.

Predictor^a^	Development cohort (CPRD Aurum) n = 5982	Validation cohort (CPRD Gold) n = 3573
Age, mean (standard deviation) year	42 (17)	43 (17)
Male sex	2940 (49.1)	1775 (49.7)
Female sex	3042 (50.9)	1798 (50.3)
Mercaptopurine equivalent dose (mg/day), median (IQR)	48.1 (36.1, 72.1)	48.1 (24.0, 72.1)
Missing	627 (10.5)	830 (23.2)
Body mass index		
<18.5 kg/m^2^	276 (4.6)	136 (3.8)
18.5–24.9 kg/m^2^	2178 (36.4)	1274 (35.7)
25.0–29.9 kg/m^2^	1562 (26.1)	955 (26.7)
≥30 kg/m^2^	1155 (19.3)	699 (19.6)
Missing	811 (13.6)	509 (14.3)
Current smoker		
No^b^	5000 (83.6)	2906 (81.3)
Yes	982 (16.4)	667 (18.7)
Alcohol use		
Non-user	1106 (18.5)	502 (14.1)
Low (1–14 units/week)	2273 (38.0)	1694 (47.4)
Moderate (15–21 units/week)	293 (4.9)	171 (4.8)
Hazardous (>21 units/week)	374 (6.3)	189 (5.3)
Ex-user	500 (8.4)	211 (5.9)
Missing	1436 (24.0)	806 (22.3)
Inflammatory conditions		
Inflammatory bowel disease	5317 (88.9)	3219 (90.1)
Rheumatoid arthritis	230 (3.8)	130 (3.6)
Systemic lupus erythematosus	243 (4.1)	109 (3.1)
Seronegative spondyloarthropathy^c^	192 (3.2)	115 (3.2)
Comorbidities		
Diabetes mellitus	457 (7.6)	240 (6.7)
Chronic Kidney Disease stage-3^d^	249 (4.2)	148 (4.1)
Drugs		
5-aminosalicylates^e^	2766 (46.2)	1730 (48.4)
Sulfasalazine	74 (1.2)	47 (1.3)
Methotrexate/Leflunomide	13 (0.2)	17 (0.5)
Statins	645 (10.8)	398 (11.1)
Allopurinol	160 (2.7)	64 (1.8)
ACE inhibitors	486 (8.1)	280 (7.8)
At least mild cytopenia or liver enzyme elevation in six-months preceding start of follow-up	785 (13.1)	529 (14.8)
Number (%) of outcome events	405 (6.8)	269 (7.5)

a Values are numbers (percentage) unless stated otherwise.

b Included non-smokers, ex-smokers, and smoking status not-available.

c Seronegative spondarthritis included psoriasis (±arthritis), ankylosing spondylitis, reactive arthritis.

d Patients with Chronic Kidney Disease stages 4 and 5 were excluded from this study.

e Included balsalazide, mesalazine, olsalazine.

### Sample size

For model development, assuming an event rate of 17 per 1000-person years from previously published studies and an average follow-up of 3.19 years, the minimum sample size needed to minimise model overfitting (a target shrinkage factor of 0.9) and ensure precise estimation of overall risk was 1748 participants (95 outcomes) for a maximum of 25 parameters, Cox-Snell R^2^ value of 0.12, a 5-year time horizon, using the formulae of Riley et al.^39^^,^^40^ The sample size for external model validation was much larger than the typically recommended minimum sample size of 200 events.

### Statistical analysis

Multiple imputation handled missing predictor data on BMI, alcohol intake, and, thiopurine dose using chained equations.^41^ Ten imputations were performed in the development dataset and five imputations in the validation dataset–a pragmatic approach considering the large size of CPRD. The imputation model included all candidate predictors, Nelson-Aalen cumulative hazard function and outcome variables. The data analysis was undertaken using the Stata command “mi estimate” in a combined dataset that included all imputations.

### Model development

Fractional polynomial regression analysis was used to model non-linear risks with continuous predictors. Nested models were employed to test whether a first-order polynomial provided a better fit than the continuous variable as a linear term. Assumptions of Cox proportional hazards model were checked using log-log plots and Schoenfeld residuals. Next, all candidate predictors were included in the Cox model and coefficients of each predictor estimated and combined using Rubin’s rule across the imputed datasets. The risk equation for predicting an individual’s risk of thiopurine discontinuation with abnormal blood-test results by 5-years was formulated using the development data. The baseline survival function at *t = 5 years (S*_*0*_*)*, where all predictor values are set to zero was estimated along with the estimated regression coefficients (β_1_x_1_ + β_2_x_2_ + … + β_p_x_p_) and the individual’s predictor values (X). This led to the equation for the predicted risk of discontinuation at 5-years of 1−S_0_(t = 5)^exp(Xβ)^.^42^

### Model internal validation and shrinkage

We bootstrapped with replacement 500 samples of the data.^43^ The full model was fitted in each bootstrap sample, with its performance quantified in the bootstrap sample (apparent performance) and the original sample (test model performance), and the optimism calculated (difference in test and apparent performance). The shrinkage for each imputation was estimated as the average of calibration slopes (test model) over all bootstrap samples. The final uniform shrinkage calculated by averaging the estimated shrinkage estimates over all imputations. Optimism-adjusted estimates of performance for the original model were calculated as the original apparent performance minus the optimism.

To account for overfitting during model development, the original β coefficients were multiplied by the final uniform shrinkage factor and the baseline hazards re-estimated conditional on the shrunken β coefficients to ensure that overall calibration was maintained. The D statistic, a measure of discrimination, interpreted as a log hazard ratio (HR), the exponential of which gives the HR comparing two groups defined by above/below the median of the linear predictor was calculated.^44^^,^^45^ R^2^ was calculated from D statistic.

### External validation

The final developed model equation was applied to the validation dataset, and calibration and discrimination were examined as above.^44^^,^^45^ Calibration of 5-year risks was examined by plotting agreement between estimated risk from the model and observed outcome risks. Predicted and observed risks were divided into 10 equally sized groups. Additionally, pseudo-observations were used to construct smooth calibration curves across all individuals via a running non-parametric smoother. Separate calibration plots were provided for each imputation. Age-group, inflammatory disease type, and whether the patient was commenced on thiopurine after the year 2010, formed the basis of sub-group analyses. Stata-MP version 16 was used for all statistical analyses and data visualisation.

This study was reported in line with the transparent reporting of a multivariate prediction model for individual prediction or diagnosis (TRIPOD) guidelines.^46^

### Health economics analysis

Our model was used to estimate the probability of outcome over a five-year period in 10 patients, one from each risk decile. They were selected by ranking the patients in terms of risk and selecting a random patient at the 5th percentile, 15th percentile, and so on until the 95th percentile (Supplementary Table S1). Patients were monitored according to each strategy for a period of five years (four years in the biennial strategy although the impact of missed abnormal blood tests spanned the five-year period). An additional monitoring appointment after cessation of treatment due to an abnormal blood test was assumed. The probability that an abnormal blood test would result in an illness because of the extended monitoring period was estimated by the clinical team members based on their experience, erring towards over estimating the risk (Supplementary Table S2). The costs and quality-adjusted life year (QALYs) losses associated with each condition was estimated following targeted literature reviews (Supplementary Table S3). Both probabilistic and deterministic analyses were performed. A monitoring appointment and blood-tests was estimated to cost £24.09 (See: Supplementary Methods). No disutility was assumed for attending a monitoring appointment.

The costs associated with monitoring and illness were estimated, as were the loss in QALYs. All values were discounted at 3.5%/annum as recommended by the National Institute for Health and Care Excellence.^47^ Results were presented in terms of incremental net monetary benefit (iNMB), assuming a cost per QALY threshold of £20,000, compared with monitoring every three months. Sensitivity analysis considered three-fold higher risks of illnesses than estimated by clinicians. Health economic data analysis and visualisation were undertaken using Microsoft Excel.

### Role of the funding source

The funder of the study had no role in study design, data collection, data analysis, data interpretation, or writing of the report. GN and AA had access to the data and had final responsibility for the decision to submit for publication.

## Results

**Fig. 1:**
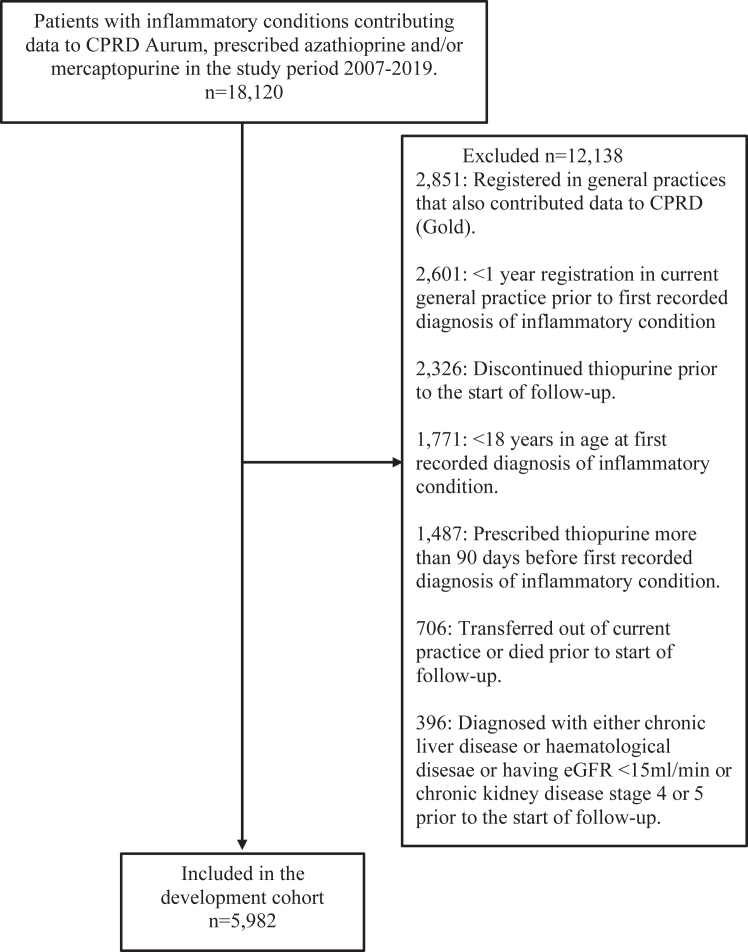
Population selection criteria for model development.

**Fig. 2:**
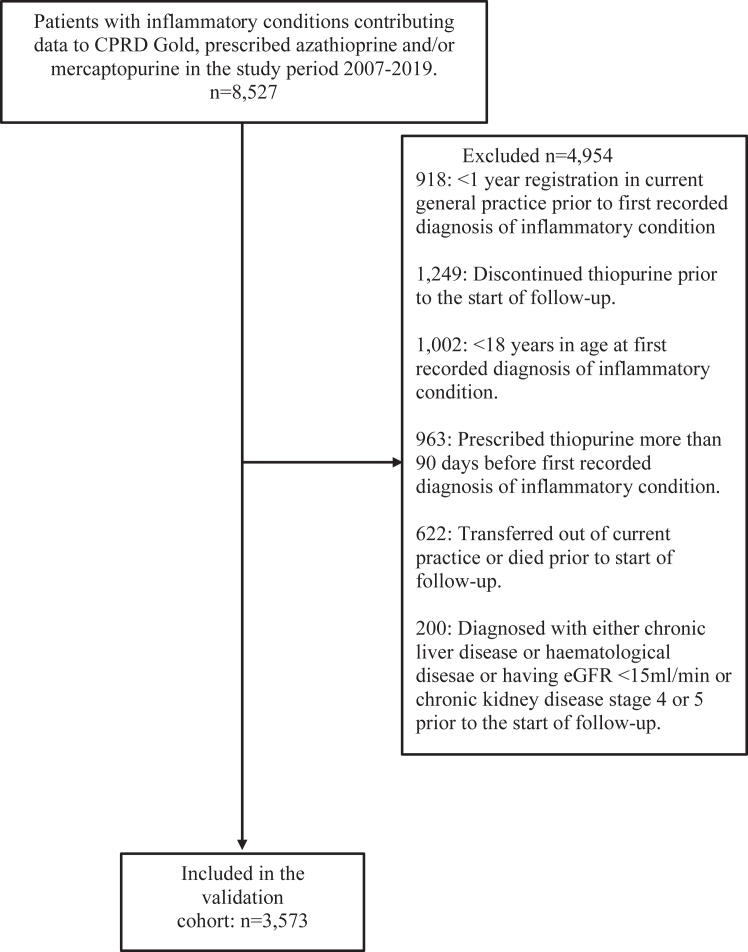
Population selection criteria for model validation.

Data for 5982 and 3573 participants that contributed 16,117 and 9075 person-years follow-up were included in the development and validation cohorts, respectively (Figs. 1 and 2). The distribution of disease and demographic factors were similar between cohorts (Table 1). The median mercaptopurine equivalent dose was 48.1 mg/day (100 mg/day azathioprine). Fourteen candidate predictors (21 parameters) were included in the model (Table 2).

**Table 2:** Final model hazard ratios and β-coefficients.

Predictors	Adjusted HR (95% CI)^a^	Coefficients
Age, years	1.01 (1.01–1.02)	0.0120654
Female sex	1.04 (0.84, 1.28)	0.0348383
Mercaptopurine equivalent dose, (mg/day)	1.00 (0.99, 1.00)	−0.0031592
Body mass index (kg/m^2^)	1.00 (0.98, 1.02)	0.0021012
Smoking status		
Non-smoker/not recorded/ex-smoker	Reference	–
Current smoker	0.98 (0.75, 1.29)	−0.0161052
Alcohol consumption		
Non-drinker	Reference	–
Low (1–14 units/week)	0.99 (0.79, 1.29)	−0.0119396
Moderate (15–21 units/week)	0.86 (0.50, 1.47)	−0.1516204
Hazardous (>21 units/week)	1.24 (0.83, 1.86)	0.215067
Ex-drinker	0.86 (0.57, 1.30)	−0.1501354
Inflammatory conditions		
Inflammatory bowel disease	Reference	–
Rheumatoid arthritis	1.56 (1.01, 2.42)	0.4467324
Systemic lupus erythematosus	2.18 (1.47, 3.24)	0.7810266
Seronegative spondyloarthropathy^b^	1.33 (0.80, 2.22)	0.2838247
Comorbidities		
Diabetes	0.91 (0.62, 1.34)	−0.0919595
Chronic kidney disease stage-3^c^	1.52 (1.05, 2.20)	0.4197865
Other immunosuppressive drugs		
None	Reference	–
5-aminosalicylates^d^	1.11 (0.89, 1.37)	0.1014151
Sulfasalazine	0.70 (0.26, 1.89)	−0.3581131
Methotrexate/leflunomide	0.89 (0.12, 6.44)	−0.1177929
Other drugs		
Statins	1.06 (0.77, 1.48)	0.0629356
Allopurinol	0.95 (0.50, 1.81)	−0.0473062
ACE inhibitors	1.05 (0.75, 1.46)	0.0440477
At-least mild cytopenia or liver enzyme elevation in six-months preceding start of follow-up	2.70 (2.17, 3.37)	0.9939685

a HR, hazard ratio; CI, confidence interval. The reported values are before shrinkage.

b Seronegative spondarthritis included psoriasis (±arthritis), ankylosing spondylitis, reactive arthritis.

c Patients with chronic kidney disease stages 4 and 5 were excluded from this study.

d Included balsalazide, mesalazine, olsalazine.

### Model development

Continuous predictors were not transformed as first-degree non-linear risk relationships were no better than linear terms (p > 0.05). Assumptions of Cox proportional hazards model were met (Supplementary Table S4, Supplementary Figure S1). 405 outcomes occurred during follow-up at an incidence (95% CI) of 25.13 (22.80–27.70)/1000 person-years. CKD, SLE, RA and either cytopenia or elevated liver enzymes during the first six months of primary-care prescription were strong predictors (Table 2). From the bootstrap, a uniform shrinkage factor of 0.80 was applied to all predictor coefficients. The final model’s cumulative baseline survival function (S_0_) was 0.938 at 5-years (Table 3). A generous number of decimal places are presented for the model coefficients. This will enable application of our model with less rounding error.

**Table 3:** Equation to predict the risk of thiopurine discontinuation with abnormal monitoring blood test results after six months of primary care prescription and within the next 5-years.

Risk score = 1−0.938^exp(0.80βX)^	
βX=	−0.0031592∗mercaptopurine equivalent daily dose
	+0.0120654∗age in years at first primary-care prescription
	+0.0348383∗female-sex
	+0.0021012∗body mass index
	−0.0161052∗current smoker
	−0.0119396∗low alcohol intake
	−0.1516204∗moderate alcohol intake
	+0.215067∗hazardous alcohol intake
	−0.1501354∗ex-alcohol intake
	+0.4467324∗rheumatoid arthritis
	+0.7810266∗systemic lupus erythematosus
	+0.2838247∗seronegative spondarthritis
	−0.0919595∗diabetes
	+0.4197865∗chronic kidney disease stage-3
	+0.1014151∗5-aminosalicylate
	−0.3581131∗sulfasalazine
	−0.1177929∗methotrexate or leflunomide
	+0.0629356∗statin
	−0.0473062∗allopurinol
	+0.0440477∗ACE-inhibitors
	+0.9939685∗at-least mild cytopenia or liver enzyme elevation within six-months of primary care thiopurine prescription.

Variables are coded 0 if absent, and 1 if present, respectively, except for mercaptopurine equivalent dose, age, and body mass index. 0.938 is the baseline survival function at 5-years and 0.80 is the shrinkage factor. Seronegative spondarthritis included psoriasis (±arthritis), ankylosing spondylitis, reactive arthritis. Patients with Chronic Kidney Disease stages 4 and 5 were excluded from this study. 5-aminosalicylate Included balsalazide, mesalazine, olsalazine. Blood test abnormality defined as either cytopenia (neutrophil count <2 × 109/l, total leucocyte count <4 × 109/l, or platelet count <150 × 109/l) or raised transaminase levels (alanine transaminase and/or aspartate transaminase >35 IU/l) during the first six months of a prescription for methotrexate in primary care.

ACE, angiotensin converting enzyme.

**Fig. 3:**
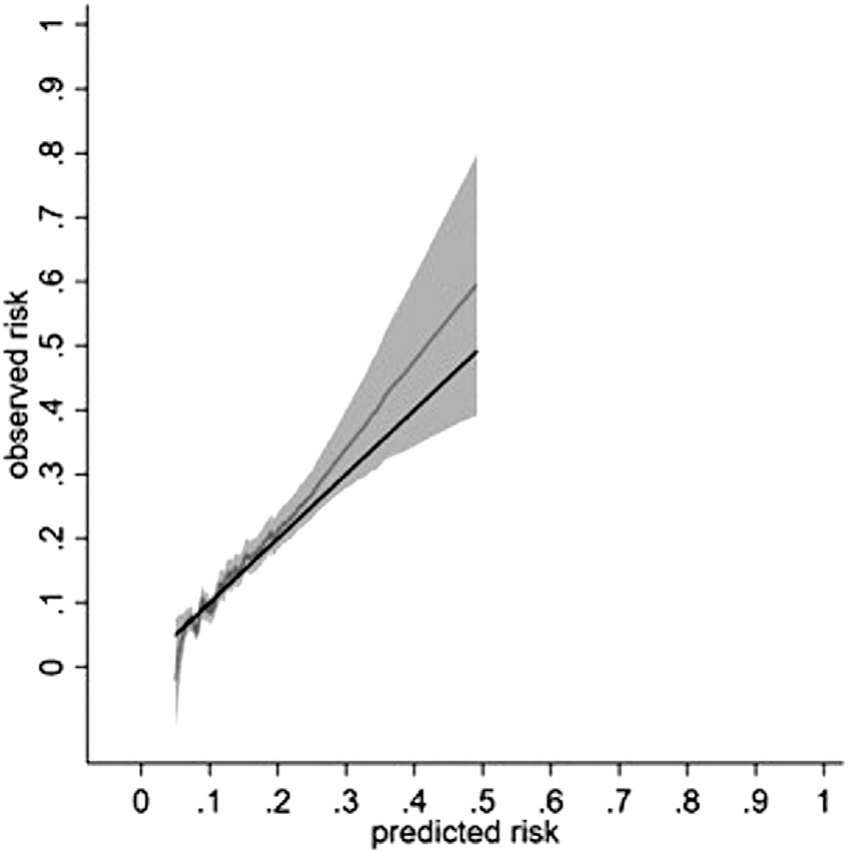
Calibration of a prognostic model for thiopurine discontinuation with abnormal monitoring blood-test results at 5 years in the development cohort. Data from a single imputed dataset was used for illustration; S_0_(t = 5) 0.938. Solid black line reflects perfect prediction.

The average model predictions matched the average observed outcome probabilities across all 10 groups of patients, with 95% CIs overlapping the 45-degree line (Fig. 3). Most patients had low risk of outcome and majority of deciles of predicted risk clustered at the lower end of the distribution (Supplementary Figure S2). The calibration curve at 5-years showed some mis-calibration at the individual level in the higher risk patients, however there were less data at the higher risk probabilities with wide 95% CIs (Fig. 3, Supplementary Figure S3). Royston *D* statistic (95% CI) was 0.91 (0.75–1.07), corresponding to HR (95% CI) 2.48 (2.12–2.92). The optimism adjusted Royston *D* statistic was 0.76 corresponding to HR 2.13 (Table 4).

**Table 4:** Model diagnostics.

Measure	Apparent performance^a^	Test performance^b^	Average optimism^c^	Optimism corrected performance^d^	Performance in external validation (CPRD Gold)
Overall calibration slope	1.00 (0.84, 1.16)	0.80 (0.64, 0.95)	0.20	0.80 (0.64, 0.96)	1.10 (0.84, 1.36)
R^2^	0.16 (0.12, 0.21)	0.14 (0.10, 0.18)	0.05	0.11 (0.07, 0.16)	0.11 (0.06, 0.16)
Royston D statistic	0.91 (0.75, 1.07)	0.84 (0.68, 0.99)	0.15	0.76 (0.60, 0.92)	0.72 (0.52, 0.92)

CPRD, Clinical Practice Research Datalink.

a Refers to performance (95% CI) estimated directly from the data that was used to develop the model.

b Determined by executing full model in each bootstrap sample (500 samples with replacement), calculating bootstrap performance, and applying same model in original sample.

c Average difference between model performance in bootstrap sample of the development dataset and performance in the development dataset.

d Obtained by subtracting average optimism from apparent performance.

### Model performance in the validation cohort

**Fig. 4:**
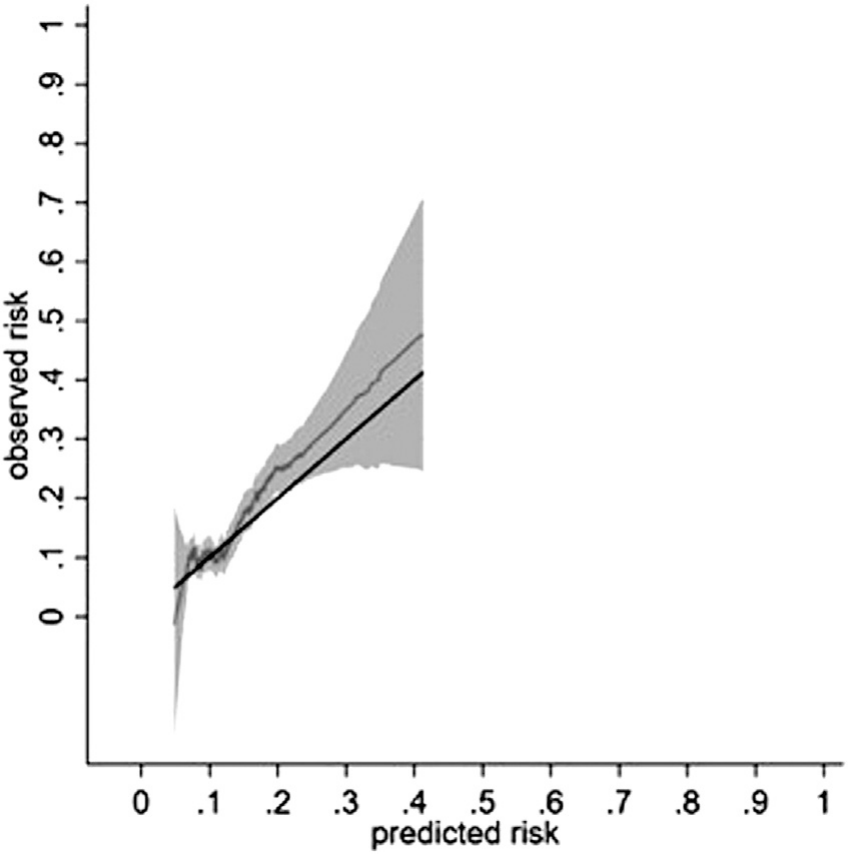
Calibration of a prognostic model for thiopurine discontinuation with abnormal monitoring blood-test results at 5 years in the validation cohort. Data from a single imputed dataset was used for illustration; S_0_(t = 5) 0.938. Solid black line reflects perfect prediction.

There were 269 outcomes incidence (95% CI) of 29.64 (26.30–33.40)/1000 person-years. The calibration slope (95% CI) across the 5-year follow-up period was 1.10 (0.84–1.36). The calibration plot showed reasonable correspondence between observed and predicted risk at 5-years across the tenths of risk (Supplementary Figure S4). Most predicted risk deciles clustered at lower end of the predicted risk distribution (Supplementary Figure S5). The smoothed calibration curve showed alignment of the predicted risk to the observed risk at low risk (Fig. 4). Model performance at years 1, 2, 3 and 4 showed a similar pattern (Supplementary Figures S6–S9). Model discrimination in the validation cohort was broadly similar to that in development cohort (Table 4). The Royston D statistic (95% CI) was 0.72 (0.52–0.92), corresponding to HR (95% CI) 2.05 (1.68–2.51). The model performed well across age-groups, in those with IBD, and in patients starting thiopurines in 2010 or later (Supplementary Figures S10 and S11).

Figs. 3 and 4 report the relationship between pseudo values of the observed risk and predicted risk via a running non-parametric smoother and therefore some values are less than zero. The pseudo values F1(t) are not constrained to fall within the range of 0–1, as they are jackknife quantitates that do not resemble individual event probabilities with the intention that E(F1(t)) = F1(t).^48^ None of the risks were less than zero when true values were used. Please see Supplementary Figures S3 and S5.

### Cost-effectiveness

**Fig. 5:**
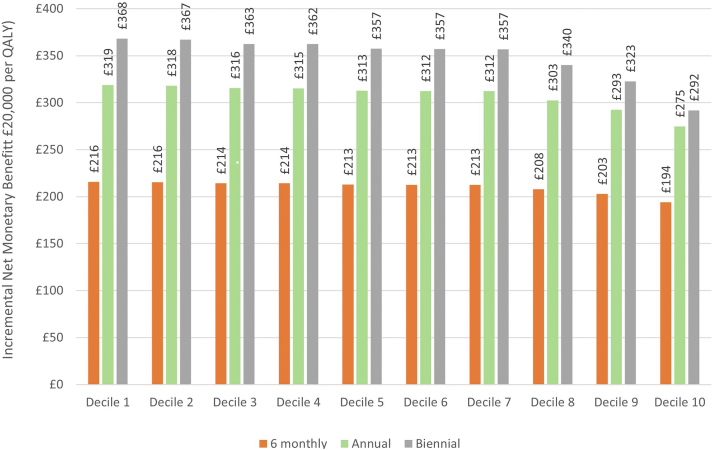
The incremental net monetary benefit associated with extended monitoring periods compared to 3 monthly monitoring across deciles of predicted risk.

All extended monitoring periods were more cost-effective than three monthly monitoring (Fig. 5). Monitoring every two years was estimated to be most cost-effective, but at higher risks the difference between annual and biennial monitoring was moderate (estimated to be an iNMB <£60 per patient in the highest-risk decile). Probabilistic analyses provided results similar to deterministic analyses and results were robust to changes in key parameters. Supplementary Figure S12 shows the results when the risks of illnesses were assumed to be triple that estimated by the clinicians. In this sensitivity analysis, biennial monitoring was estimated to be most cost-effective up to decile 7, with annual monitoring most cost-effective for higher-risk deciles; all extended monitoring periods remained more cost-effective than 3-monthly monitoring. Disaggregated results for the base case are shown in Supplementary Table S5, with combined results in Supplementary Table S6.

## Discussion

We have developed and externally validated a prognostic model for clinically-significant thiopurine toxicity detected on monitoring blood-tests during long-term treatment. Our prognostic model performed well in predicting outcomes for up to five-years with excellent calibration and discrimination and in clinically relevant subgroups. It was cost-effective to increase the interval between monitoring blood-tests.

Target organ toxicity can occur several years after starting on thiopurines e.g., hepatotoxicity due to late metabolic shunting to 6-methylmercaptopurine ribonucleotide (6-MMPR) production occurred in 1.7% patients prescribed thiopurines after median 644 days of treatment.^49^ Another cohort study with long follow-up showed that adverse effects occur later in therapy, but are far less frequent in this period.^11^ It is in this later period that we propose our risk score has its utility. The risk-score output from the model may permit individualised monitoring strategies during long-term thiopurine treatment, with patients at low risk of toxicity undergoing less frequent blood-tests e.g., six monthly or annually. These findings should be considered by specialty guideline writing groups in order to modify their monitoring recommendations.

Nevertheless, regardless of the frequency of monitoring blood-tests undertaken, blood-test abnormalities should be should be acted upon as clinically appropriate. Due vigilance needs to be maintained for hepatic nodular regenerative hyperplasia which is indicated by thrombocytopenia and/or elevated liver enzymes and non-cirrhotic portal hypertension which may also manifest with anaemia, splenomegaly, variceal bleed, and/or ascites.^50^^,^^51^ The median time from starting azathioprine to hepatic nodular regenerative hyperplasia was 48 months in a large case-series.^50^

While our results indicate that the interval between monitoring blood-tests undertaken to screen for asymptomatic toxicity may be increased for the vast majority of patients prescribed thiopurines, blood-tests are an integral part of disease activity assessment in patients with many inflammatory conditions such as inflammatory bowel disease and systemic lupus erythematosus. Such blood tests should be undertaken when clinically indicated. For instance, it may be necessary to review a patient with active disease every three to six months with an updated full blood count, liver function tests, and urea, electrolytes and creatinine measurement in order to assess disease activity.

We did not have data on the date of first thiopurine prescription in the hospital clinic. As it typically takes four to six months to stabilise a patient on adequate dose of thiopurines in the UK, the model may be used to risk-stratify monitoring after six months of shared-care prescription or after one year from first thiopurine prescription in healthcare systems without shared care prescribing. As reported previously, increasing age, and prior blood-test abnormalities were strong predictors of thiopurine toxicity in the current study.^52^^,^^53^ The reduced thiopurine clearance in CKD and occurrence of renal involvement in SLE could also explain their being strong predictors of thiopurine toxicity in this study.^12^, ^54^

Genomic studies have established thiopurine methyl transferase (TPMT) activity prior to thiopurine prescription as a useful and cost-effective predictor of myelotoxicity, and though it has a clear place, TPMT testing cannot predict all myelosuppression.^4^^,^^55^, ^56^, ^57^ Unfortunately, the results of TPMT testing are not available in the CPRD and we were unable to include them in the prognostic model. For therapeutic drug monitoring (TDM) there is uncertainty on how well it predicts future toxicity with 6-thioguanine nucleotides (6-TGN) and 6-MMPR. At treatment start TDM did not perform well in predicting hepatotoxicity and myelotoxicity in two studies but was a strong predictor of myelotoxicity in the TPOIC trial cohort.^58^, ^59^, ^60^ Another study reported more cases with leucopenia in the presence of high 6-TGN levels compared to low 6-TGN levels although the differences were not statistically significant.^61^ Reactive TDM during thiopurine treatment aids accurate ascertainment of toxicity, facilitates dose-reduction for dose-dependent side effects, and allows personalised dosing, thereby improving persistence on treatment and disease outcomes.^62^^,^^63^ TDM for thiopurines is not commonly performed in the UK and their results are not available in the CPRD. Further research is required to explore if the inclusion of TPMT, and TDM biomarkers at baseline would improve model performance. Future validation studies in Asian and Hispanic populations should also consider including polymorphisms in the nucleoside diphosphate-linked moiety X-type motif (NUDT) 15 in the model as these polymorphisms are common in the Asian and Hispanic populations with a prevalence of 27%.^64^

There are several strengths of this study. We used a large real-world and nationally representative dataset for model development and a similar independent dataset for external validation allowing our results to have a high precision.^14^^,^^15^ We included a range of inflammatory diseases giving broad generalisability to the results. The outcome required the blood-test abnormality to be associated with thiopurine discontinuation, thereby predicting clinically relevant outcomes rather than minor or transient variations in blood parameters. Our health economic analysis provides evidence of cost efficacy for alternate monitoring strategies that were robust to changes in assumptions. Finally, as it is derived directly from available general practice data, the prognostic model could easily be built into GP electronic health records (e.g., as a calculator) to simplify its use.

However, several limitations of the study ought to be considered. First, we did not have data on concurrent use of biologics as these are hospital prescribed in the UK. However, there is no evidence that biologics increase the forms of thiopurine toxicity for which monitoring blood-tests are recommended.^65^^,^^66^ Second, the results of TPMT testing is not available in the CPRD. We do not believe that this is a major limitation as approximately 90% of Whites are not deficient in TPMT, and the vast majority of myelotoxicity due to TPMT deficiency occurs in the first few months of treatment.^67^^,^^68^ Participants included in our study were already prescribed thiopurines for at-least six months from primary-care after an initial period of prescription and monitoring from hospital outpatient clinic and most instances of myelotoxicity from thiopurine deficiency would have occurred before follow-up started and this prognostic model should not be used to replace the need for TPMT testing. Furthermore, the model performance was comparable in the years 2010 and after when TPMT testing became more widespread in the UK and in the entire cohort. Third, low numbers in the highest risk groups resulted in uncertainty regarding predictions for these groups. Fourth, the use of UK primary-care data for both model development and validation imply that further validation in other geographic regions may be required. Fifth, it might be argued that not performing competing risk regression is a weakness of our methodology, however, as there were very few (0.07%) deaths in the development cohort and no deaths in validation cohort during the 5-year follow-up period we do not believe this limits the validity of our findings. Sixth, patients that were prescribed thiopurines exclusively by a hospital specialist due to complex comorbidities and/or being at very high risk of toxicity were not included in this study. The prognostic model should not be used in this population. This is not a significant limitation as it is unusual to have such hospital prescribing and monitoring of thiopurines. Finally, as patients prescribed thioguanine were not included in this study, the results should not be extrapolated to this drug.

In conclusion, we developed an easy to use prognostic model for thiopurine discontinuation with abnormal monitoring blood-test results that may be used in clinical practice. It was cost-effective to monitor less frequently than is currently recommended. These findings should be of interest to guideline writing groups when considering recommendations upon the frequency of monitoring blood-tests during long-term thiopurine treatment.

## Contributors

GN, MJG, HCW, TC, MWT, GPA, CPF, CDM, DAW, MDS, RDR, and AA designed the study. GN analysed the data supervised by MJG, RDR, and AA. GN and AA accessed and verified the underlying data. MDS performed health-economic analysis. GN, MJG, HCW, TC, MWT, GPA, CPF, CDM, DAW, MDS, RDR, and AA interpreted the data. AA and GN drafted the original submitted manuscript. AA led on the revision of the manuscript after peer review. All authors critically evaluated and revised the manuscript and approved the final version. The corresponding author attests that all listed authors meet authorship criteria and that no others meeting the criteria have been omitted. AA is the guarantor.

## Data sharing statement

Data used in the study are from the Clinical Practice Research Datalink (CPRD). Due to CPRD licencing rules, we are unable to share data used in this study with third parties. The data used in this study may be obtained directly from the CPRD. Study protocol is available from www.cprd.com.

## Declaration of interests

A.A. has received Institutional research grants from AstraZeneca and Oxford Immunotech; and personal fees from UpToDate (royalty), Springer (royalty), Cadilla Pharmaceuticals (lecture fees), NGM Bio (consulting), Limbic (consulting) and personal fees from Inflazome (consulting) unrelated to the work. GPA has received consulting fees from Abbott Products, Albireo Pharma, Amryth, AstraZeneca, BenevolentAI Bio, Clinipace, DNDI, GlaxoSmithKline, Merck, NuCANA, Puretech, Pfizer, Roche Diagnostics, Servier Pharmaceuticals, W.L. Gore & Associates paid to the University of Nottingham unrelated to the work. CPF has received Consultancy/Advisory board fees from Abbvie, GenMab, Incyte, Morphosys, Roche, Takeda, Ono, Kite/Gilead, BMS/Celgene, BTG/Veriton and departmental research funding from BeiGene unrelated to the work. The other authors have no conflict of interest to declare. Keele University has received research funding for CDM from NIHR, MRC, Versus Arthritis and BMS. CDM is Director of the NIHR School for Primary Care Research. HCW worked for the National Institute of Health Research 2015–2021. He had no part to play in the funding of this study.
