# Supplementary material for: Risk-stratified monitoring for thiopurine toxicity in immune-mediated inflammatory diseases: prognostic model development, validation, and, health economic evaluation
Source: eClinicalMedicine. 2023 Sep 14;64:102213. doi: 10.1016/j.eclinm.2023.102213 (PMC10514402; doi:10.1016/j.eclinm.2023.102213)
Supplement: Supplementary Material [file mmc1.pdf]

Contents

Health economics supplementary methods: 3

Supplementary Table 1: Individual patient's characteristics at the midpoint of each decile. 5

Supplementary Table 2: The estimated probability that a delayed abnormal blood test would have caused a more serious condition. 6

Supplementary Table 3: The estimated costs and Quality Adjusted Life Years (QALY) losses associated with each condition derived from the literature [1-11]. 7

Supplementary Table 4: Cox proportional hazard assumption global test results 8

Supplementary Table 5: Disaggregated results in the base case 9

Supplementary Table 6: Aggregated Results in the base case compared with current 3-month monitoring 10

Figure S1: Cox proportional hazard assumption: log-log plots for individual covariates. 11

Figure S2: Distribution of predicted risk in the model derivation cohort at 5 years 18

Figure S3: Calibration of a prognostic model for thiopurine discontinuation with abnormal monitoring blood-test results at 5-years in the development cohort 19

..... 19

Figure S4: Distribution of predicted risk in the model validation cohort at 5 years 20

Figure S5: Calibration of a prognostic model for thiopurine discontinuation with abnormal monitoring blood-test results at 5-years in the validation cohort 21

Figure S6: Calibration of a prognostic model for thiopurine discontinuation with abnormal monitoring blood-test results at 1 year in the validation cohort 22

Figure S7: Calibration of a prognostic model for thiopurine discontinuation with abnormal monitoring blood-test results at 2 years in the validation cohort 23

Figure S8: Calibration of a prognostic model for thiopurine discontinuation with abnormal monitoring blood-test results at 3 years in the validation cohort 24

Figure S9: Calibration of a prognostic model for thiopurine discontinuation with abnormal monitoring blood-test results at 4 years in the validation cohort 25

Figure S10: Calibration of a prognostic model for thiopurine discontinuation with abnormal monitoring blood-test results at 5 years in the validation cohort: stratified according to age. 26

Figure S11: Calibration of a prognostic model for thiopurine discontinuation with abnormal monitoring blood-test results at 5 years in patients who initiated treatment in 2010 or later and IBD: validation cohort. 27

Figure S12: Incremental net monetary benefit results when the risks of serious conditions estimated by clinicians were tripled 28

Supplementary references 29

Health economics supplementary methods:

Derivation of the estimates contained in Supplementary Table 3.

All costs were inflated to 20/21 prices where required using Jones and Burns (1).

Acute kidney injury (AKI) The estimated cost for AKI used a weighted cost for the following NHS reference currency codes: LA07H, LA07J, LA07K, LA07L, LA07M, LA07N, and LA07P. This provided a value of £1961, which was inflated to a value of £2022. Data on the utility or QALY loss associated with AKI was redacted in NICE TA755 (2) and so the QALY loss were estimated in an alternative manner. The number of days in hospital due to AKI was estimated by dividing £1961 by £378 (the average cost per regular day or night admission) which equalled 5·19. The QALY loss was assumed to be equivalent of five days with a loss of utility of 1, assuming that this captures QALY loss prior to hospitalisation and after discharge. This results in an estimated QALY loss of 0.01

Acute liver failure (ALF) The cost for ALF in patients who were hospitalised was estimated using the following NHS reference currency codes: GC01C, GC01D, GC01E, and GC01F, which resulted in a cost of £2461. The cost of liver transplantation was taken from GA15A assuming all patients were aged 18 years or over (£21,546), with ongoing annual maintenance costs after a liver transplant, assumed to last for a maximum of 20 years, taken from NICE TA755 (£5949) (2). The proportion of patients who were hospitalised following ALF (53%) the proportion that require a liver transplant (8%) and those that died (5%) were taken from Andrade *et al (3)*. Those who died or had a transplant were also assumed to incur the costs of an ALF hospitalisation stay. Taking the outcomes into account and inflating to 20/21 prices the weighted cost for patients having ALF was estimated to be £3352. The estimated QALY loss associated with ALF was calculated as the addition of the estimated losses associated with hospitalisation, transplant and death. For hospitalisation, it was assumed that the utility of a patient was reduced to zero for a period of a week. For transplant, it was assumed that the utility in the year of a transplant was 0·45 and then 0·67 in subsequent years as reported in NICE TA507 (4) estimating a utility decrement compared with the average utility of a 60-year-old (0·816) of 0·366 in year one, and 0·146 in subsequent years. It was assumed that this decrement would last for a period of 19 years.

For patients who die, the QALY loss associated with a death from sepsis reported in Stevenson *et al.* (5) of 11·32 was used. Combining these components and considering the proportion of patients experiencing each event resulted in an estimated QALY loss of 0·651.

Anaemia The costs for anaemia were taken from NICE TA507(4) which provided a managements cost of £369·57 and four weeks of erythropoietin treatment. When inflated to 20/21 prices the estimated cost in £465. For QALY losses, it was assumed that there would be the equivalent of half a day with a utility loss of 1.

Chronic Kidney Disease (CKD) The costs for a change in CKD stage was taken from NICE TA755 (2)assuming a change from CKD stage 3 to CKD stage 4, which reported an increase cost per year of £3030. It was assumed that the relative increase in stage would last for a period of 10 years, resulting in a discounted cost increase of £26,083. The utility loss per CKD stage was redacted in NICE TA755, but it was reported that these were similar to utilities reported in NICE TA599 (6), which indicated that there was a 0.152 utility loss between CKD stage 4 and CKD stage 3. Assuming that the increase in stage would last for a period of 10 years, results in a discounted QALY loss of 1·31.

Cirrhosis NICE TA507 (4) reports that the combined initial evaluation costs and monitoring costs over the first 24 weeks were £2176 with annual costs of patients with compensated cirrhosis being £1138. Assuming that these increased costs were relevant for a 20-year period and inflating to a price year of 20/21 results in increased costs of £21,700. The utility loss associated with cirrhosis was assumed to be 0·2 based on values used in NICE TA507 (4) which were 0·79 for non-cirrhotic patients with sustained virologic response and 0·59 for patients with compensated cirrhosis with sustained virologic response. Assuming a 0·2 utility decrement for a period of 20 years results in an estimated discounted QALY loss of 3·04.

Drug-induced liver injury (DILI) In the absence of better evidence, it was assumed that the costs and QALY losses associated with DILIs were equal to those associated with ALF.

Early Fibrosis The utility loss associated with early fibrosis was taken from NICE Clinical Guideline 49 (7) which suggested that a utility loss of 0·03 was associated with non-treated non-alcoholic steatohepatitis. This value was believed likely to be an overestimate in the public discussion of MRI-based technologies for the assessment of people with non-alcoholic fatty liver disease (8). No costs were assumed to be associated with early fibrosis.

Low neutrophil count plus sepsis The estimated cost for low neutrophil count plus sepsis was the weighted average of NHS reference costs associated with sepsis with no, or single, interventions (currency codes WJ06D, WJ06E, WJ06F, WJ06G, WJ06H, and WJ06J). This was a value of £2313 when inflated to 20/21 prices. The QALY loss associated with sepsis was comprised of a death component and a chronic disutility component. For death it was assumed, from Stevenson *et al.* (5) that 13% of patients with sepsis would die, each losing 11·32 QALYs. For patients who didn't die it was assumed that there would be a utility loss of 0·136, estimated by subtracting 0·68 (the utility post-sepsis from Stevenson *et al.*) from the average utility of a 60-year-old (0·816). Assuming this disutility lasted for 10 years, there would be a discounted QALY loss of 1·02. Adding the utility losses, weighted by the probability of dying resulted in an average QALY loss of 2·49 per person.

Neutropenic sepsis The cost for neutropenic sepsis was the weighted average of NHS reference costs associated with sepsis with multiple interventions (currency codes WJ06A, WJ06B, and WJ06C). This was a value of £8558 when inflated to 20/21 prices. In addition, a cost of £898 was incurred for filgrastim use resulting in a total cost of £9456. It was assumed that the QALY losses for neutopenic sepsis was the same as for low neutrophil count plus sepsis.

Thrombocytopenia requiring hospitalisation The cost for thrombocytopenia was the weighted average of NHS reference costs associated with thrombocytopenia treatment (currency codes SA12G, SA12H, SA12J, and SA12K) which was £771. Inflating this cost and adding £223 for a platelet infusion taken from Item Code BC045 from NHS Blood and Transplant prices (9), resulted in an overall cost of £1018. The QALY loss was assumed to be equivalent of six days with a loss of utility of 1, based on six days in hospitalisation using immune thrombocytopenic purpura as a proxy for thrombocytopenia requiring hospitalisation (10) and assuming that this captures QALY losses prior to hospitalisation and after discharge. This results in an estimated QALY loss of 0.016.

Thrombocytopenia with superficial bleeding. This condition was assumed to be associated with an outpatient appointment cost, which was taken from NHS Reference costs and inflated to 20/21 prices resulting in a cost of £137.

Cost of monitoring blood-tests The cost estimated for a monitoring appointment was based on 10 minutes of phlebotomist time (costed as band 4) at £5·83 (1) ; 5 minutes of GP time at £13·17 including qualification costs (1); £2·61 for a full blood count, £1·24 for a liver function test (11) and £1·24 for a creatinine test (which was assumed to have the same price as a liver function test, a renal function test and a calcium phosphate test) (11).

Supplementary Table 1: Individual patient's characteristics at the midpoint of each decile.

Decile	Age (yr.)	Sex	TP dose (MP/d)	BMI (kg/m^2)	Current Smoker	Alcohol Consumption	Disease	DM	CKD	IS drug	Statins	Allopurinol	ACE inhibitors	BTA
1	29	F	96·154	22·1	No	Low	IBD	No	No	No	No	No	No	No
2	20	F	48·077	17·2	No	Non-drinker	IBD	No	No	No	No	No	No	No
3	24	F	48·077	26·7	No	Low	IBD	No	No	5-ASA	No	No	No	No
4	44	F	96·154	31·0	No	Non-drinker	IBD	No	No	No	No	No	No	No
5	41	F	12·019	23·3	No	Low	IBD	No	No	No	No	Yes	No	No
6	48	M	11·015	25·6	Yes	Non-drinker	IBD	Yes	No	No	No	No	No	No
7	54	M	48·077	23·8	No	Moderate	IBD	No	No	5-ASA	No	No	No	No
8	63	F	6·976	26·0	No	Low	IBD	No	No	5-ASA	No	No	No	No
9	19	F	48·077	25·0	No	Low	IBD	No	No	5-ASA	No	No	No	Yes
10	61	M	72·115	30·9	No	Low	IBD	No	No	5-ASA	No	No	Yes	Yes

5- ASA: - 5-aminosalicyclic acid; MP: - Mercaptopurine; BMI: - Body Mass Index; BTA: - Blood Test abnormalities within 6 months of primary care thiopurine prescription; CKD: - Chronic Kidney Disease; DM: - diabetes mellitus; F: - female; IBD: - Inflammatory Bowel Disease; IS: – immunosuppressive drug; M: - male; TP: - thiopurine

Supplementary Table 2: The estimated probability that a delayed abnormal blood test would have caused a more serious condition.

Adverse Event	6-monthly monitoring	Annual monitoring	Two-yearly monitoring
Acute kidney injury	0·0001	0·0001	0·0001
Acute liver failure*			
Anaemia	0·0001	0·0002	0·0004
Chronic kidney disease	0·0001	0·0001	0·0004
Cirrhosis*			
Drug-induced liver injury	0·001	0·001	0·001
Early fibrosis*			
Low neutrophil count plus sepsis	0·001	0·002	0·005
Neutropenic sepsis	0·0001	0·0002	0·0004
Thrombocytopenia requiring hospitalisation*			
Thrombocytopenia with superficial bleeding*			

*The clinical experts did not think that thiopurine causes these outcomes.

Supplementary Table 3: The estimated costs and Quality Adjusted Life Years (QALY) losses associated with each condition derived from the literature [1-11].

Condition	Costs (£)	QALY loss
Acute Kidney Injury	2022	0·014
Acute Liver Failure	3352	0·651
Anaemia	465	0·001
Chronic Kidney Disease	26,083	1·308
Cirrhosis	21,700	3·042
Drug-Induced Liver Injury	3352	0·651
Early Fibrosis	0	0·075[†]
Low neutrophil count plus sepsis	2313	2·490
Neutropenic Sepsis	9456	2·490
Thrombocytopenia requiring hospitalisation	1018	0·016
Thrombocytopenia with superficial bleeding	133	0·001

[†]per 6 months unidentified

Supplementary Table 4: Cox proportional hazard assumption global test results

Predictors	rho	chi2	df	Prob>chi2
Mercaptopurine equivalent dose, (mg/day)	0.00288	0	1	0.9527
Age (years)	0.01351	0.08	1	0.7759
Sex	-0.05127	1.13	1	0.2883
Body mass index (kg/m2)	-0.03256	0.4	1	0.525
Current smoker	-0.04725	0.91	1	0.3389
Low alcohol drinker (1-14 units/week)	0.11933	5.76	1	0.0164
Moderate alcohol drinker (15-21 units/week)	0.0388	0.62	1	0.4305
Hazardous alcohol drinker (>21 units/week)	0.00615	0.02	1	0.9017
Ex-drinker	0.0359	0.5	1	0.4806
Inflammatory bowel disease	-0.03567	0.51	1	0.4744
Rheumatoid arthritis	-0.00565	0.01	1	0.9083
Systemic lupus erythematosus	0.04818	0.96	1	0.3284
Diabetes	-0.0353	0.44	1	0.5067
Chronic Kidney Disease stage-3	-0.06007	1.55	1	0.2135
5-acetyl salicylates (Balsalazide, mesalazine and olsalazine)	-0.02377	0.23	1	0.6298
Sulfasalazine	-0.02281	0.21	1	0.6473
Methotrexate/leflunomide	-0.02581	0.27	1	0.6051
Statins	0.00403	0.01	1	0.937
Allopurinol	-0.0055	0.01	1	0.9111
ACE inhibitors	-0.03329	0.43	1	0.5108
At-least mild cytopenia or liver enzyme elevation in six-months preceding start of follow-up	-0.10306	4.25	1	0.0392
**Global test**		**20.18**	**21**	**0.5098**

Data from a randomly selected imputation.

Supplementary Table 5: Disaggregated results in the base case

	Monitoring Appointments				Monitoring Costs Saved compared with current 3-month monitoring (£)			Abnormal blood results identified late compared with current 3-month monitoring			Costs associated with late identification of abnormal blood results (£)			QALY losses associated with late identification of abnormal blood results		
Decile	3-months	6-month	Annual	Biennial	6-month	Annual	Biennial	6-month	Annual	Biennial	6-month	Annual	Biennial	6-month	Annual	Biennial
1	19·29	9·67	4·85	1·98	216·99	325·52	386·90	0·037	0·052	0·061	0·26	0·62	1·70	0·0001	0·0003	0·0007
2	19·27	9·65	4·84	1·98	216·70	325·09	386·38	0·038	0·053	0·063	0·26	0·64	1·76	0·0001	0·0003	0·0007
3	19·17	9·61	4·82	1·97	215·60	323·43	384·41	0·043	0·060	0·072	0·30	0·73	1·99	0·0002	0·0004	0·0008
4	19·17	9·61	4·82	1·97	215·53	323·33	384·29	0·043	0·061	0·072	0·30	0·73	2·00	0·0002	0·0004	0·0009
5	19·06	9·55	4·80	1·97	214·31	321·50	382·10	0·049	0·068	0·081	0·34	0·82	2·26	0·0002	0·0004	0·0010
6	19·05	9·55	4·80	1·97	214·23	321·39	381·97	0·049	0·069	0·082	0·34	0·82	2·26	0·0002	0·0004	0·0010
7	19·05	9·55	4·80	1·97	214·15	321·27	381·82	0·049	0·069	0·082	0·34	0·83	2·29	0·0002	0·0004	0·0010
8	18·69	9·38	4·72	1·96	209·98	315·04	374·37	0·068	0·095	0·113	0·47	1·15	3·14	0·0002	0·0006	0·0013
9	18·31	9·20	4·64	1·94	205·65	308·55	366·60	0·087	0·122	0·144	0·60	1·47	4·02	0·0003	0·0008	0·0017
10	17·63	8·88	4·49	1·92	197·83	296·85	352·61	0·120	0·169	0·200	0·84	2·04	5·57	0·0004	0·0011	0·0024

Supplementary Table 6: Aggregated Results in the base case compared with current 3-month monitoring

	Net Cost Savings (£)			Net QALY losses		
Decile	6-month	Annual	Biennial	6-month	Annual	Biennial
1	216·74	324·90	385·20	0·0000	0·0003	0·0008
2	216·44	324·44	384·62	0·0000	0·0003	0·0009
3	215·30	322·71	382·42	0·0001	0·0004	0·0010
4	215·23	322·60	382·28	0·0001	0·0004	0·0010
5	213·97	320·68	379·84	0·0001	0·0004	0·0011
6	213·89	320·57	379·69	0·0001	0·0004	0·0011
7	213·81	320·44	379·54	0·0001	0·0004	0·0011
8	209·51	313·89	371·23	0·0001	0·0006	0·0016
9	205·04	307·08	362·59	0·0001	0·0008	0·0020
10	197·00	294·81	347·04	0·0001	0·0010	0·0028

Figure S1: Cox proportional hazard assumption: log-log plots for individual covariates. Data from a randomly selected single imputation were used.

A: Sex

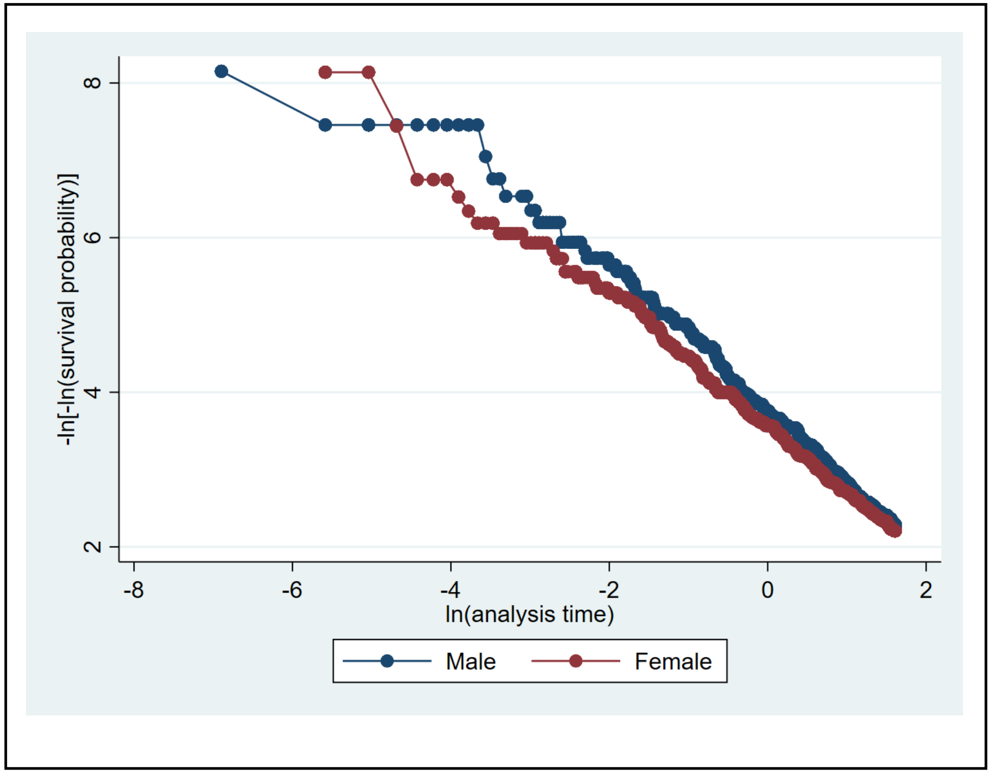

B: Current smoking status

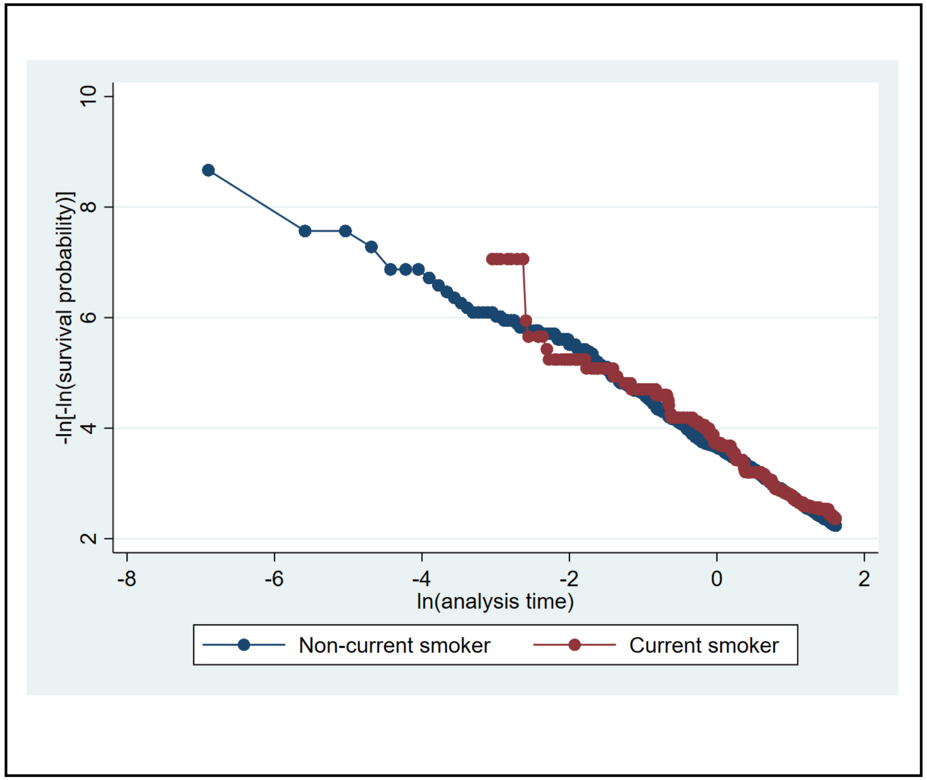

C. Alcohol consumption

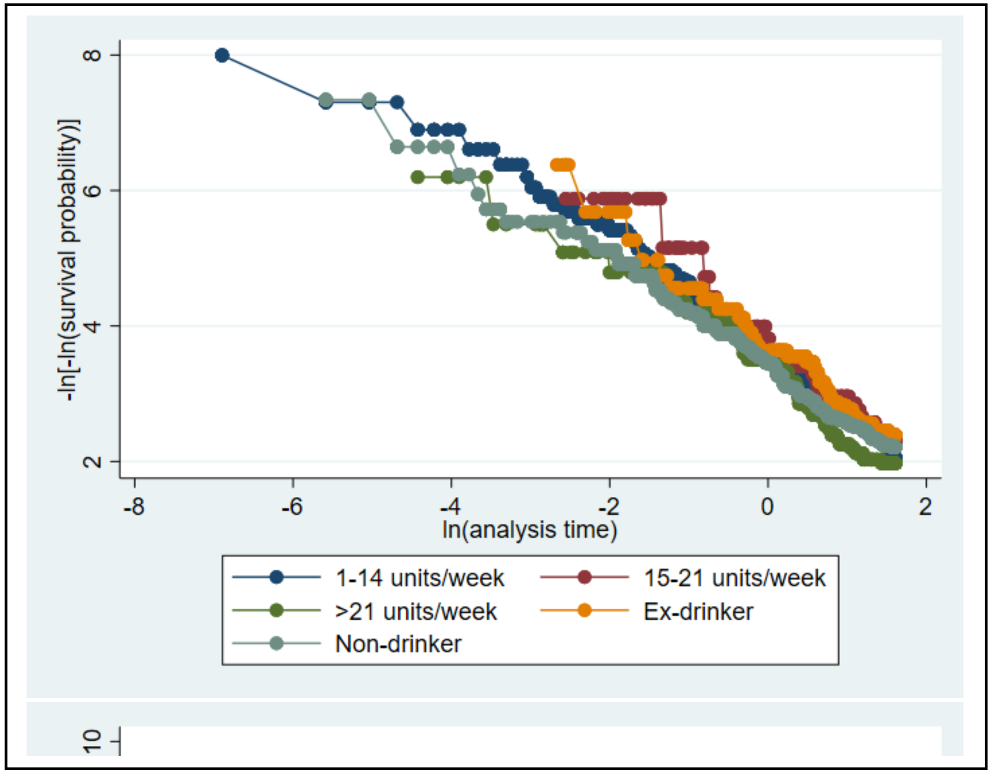

D. Inflammatory condition type

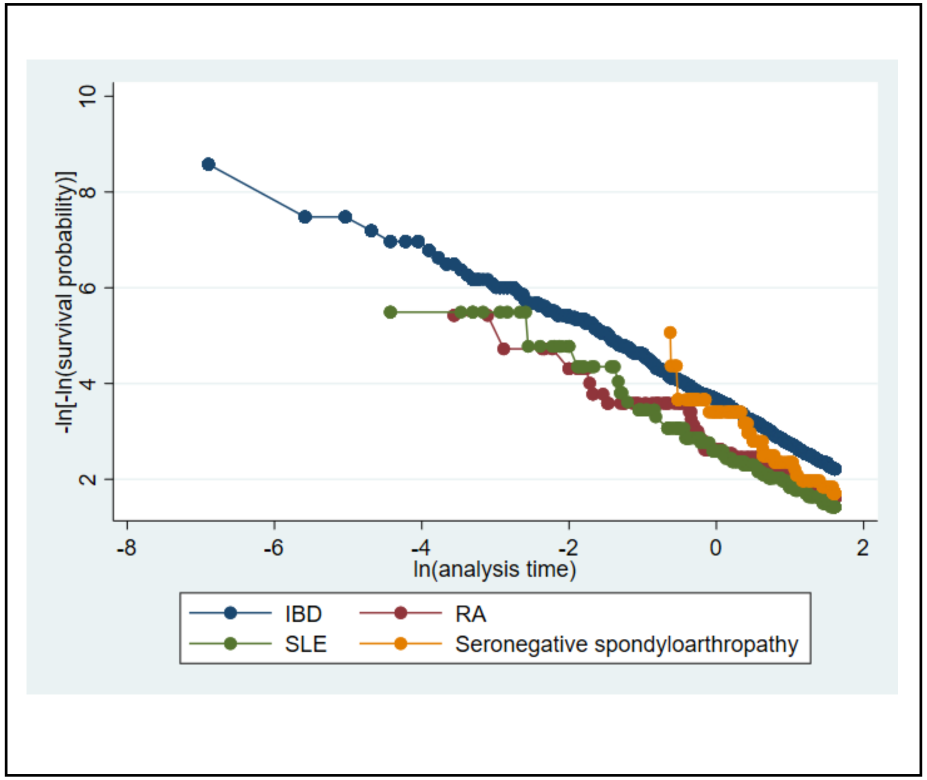

E. Other immune suppressing drug

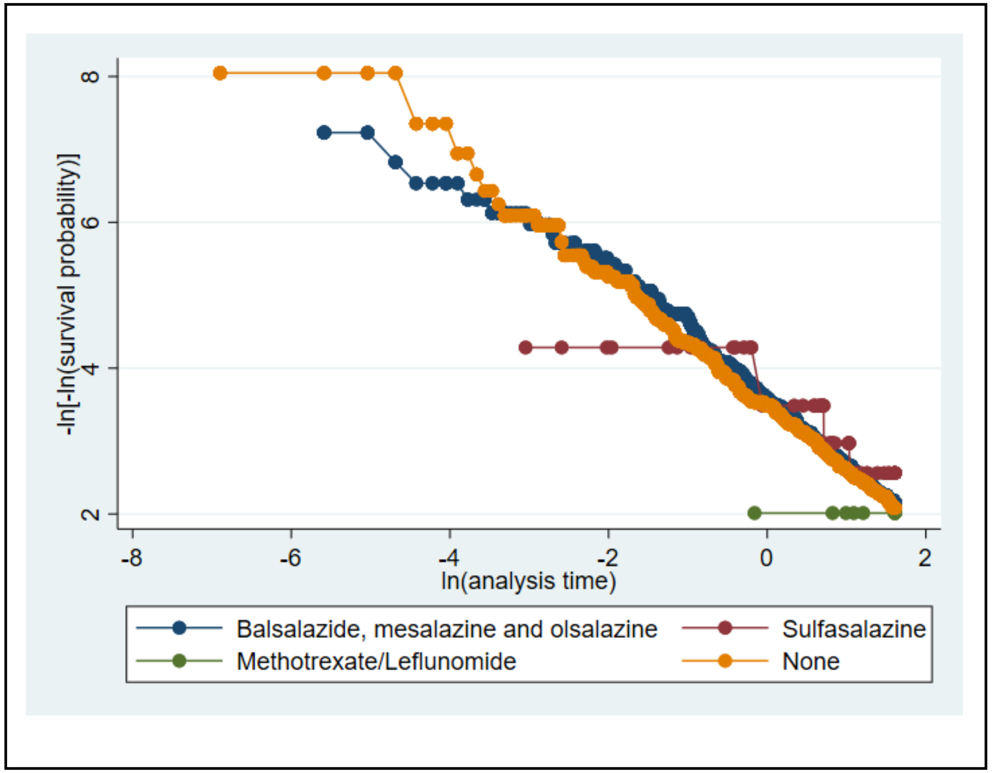

F. Diabetes

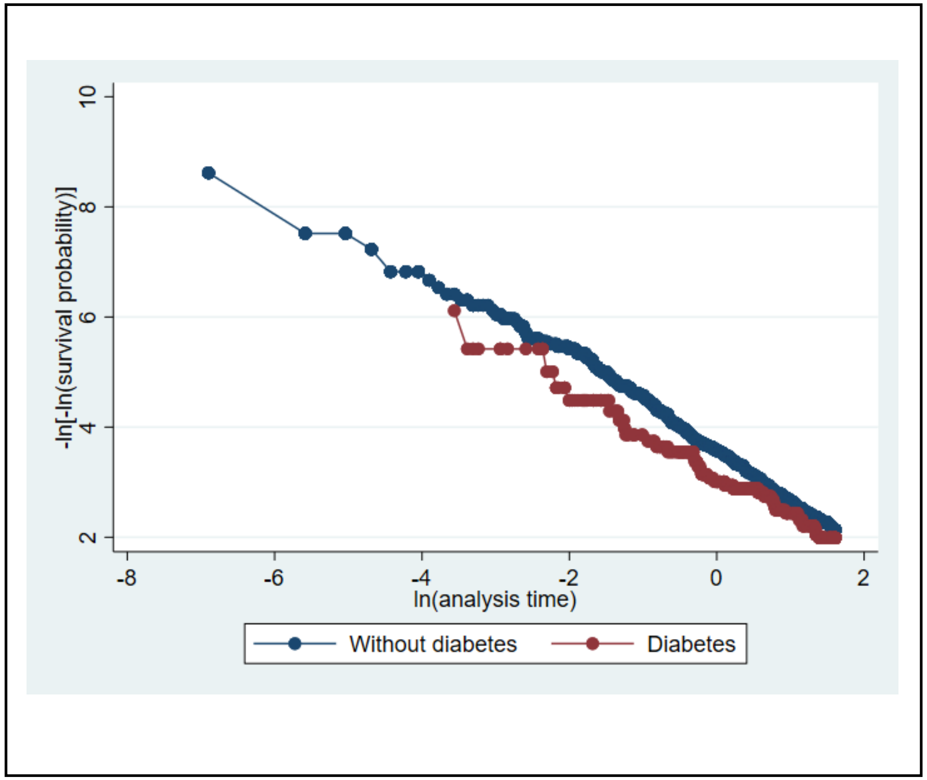

G. Chronic Kidney Disease

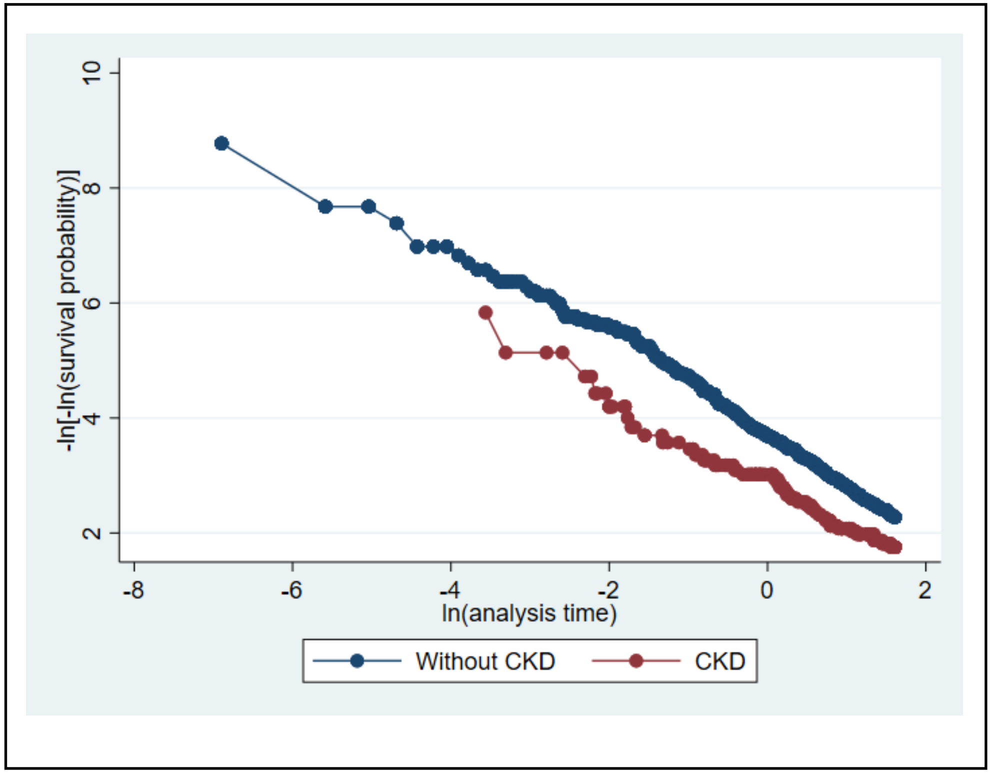

H. Statins

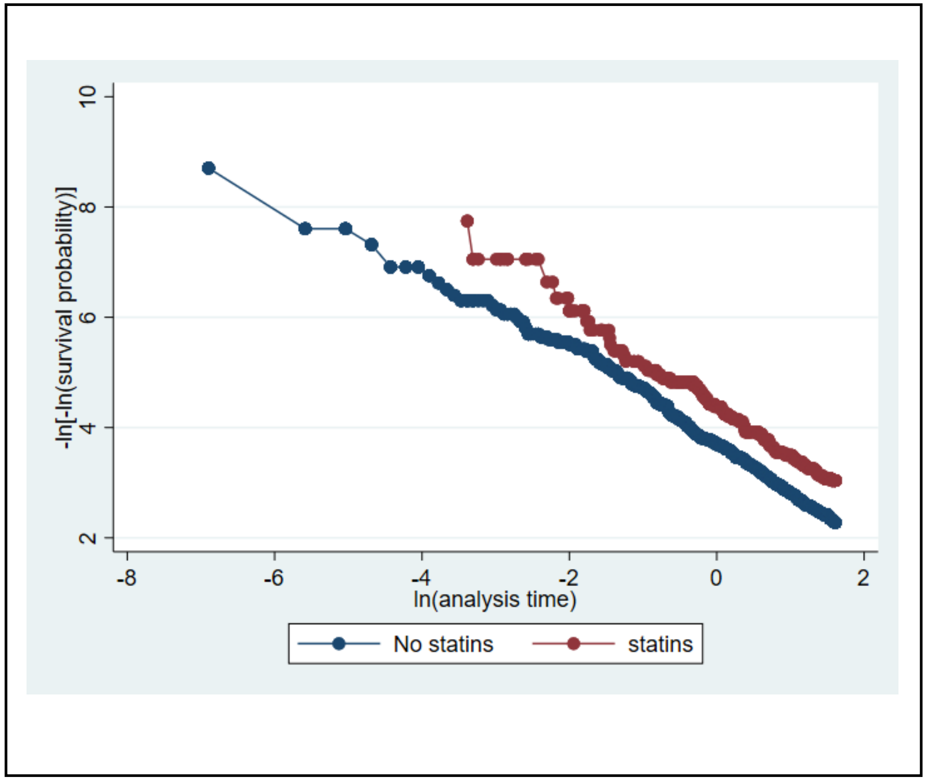

I. Allopurinol

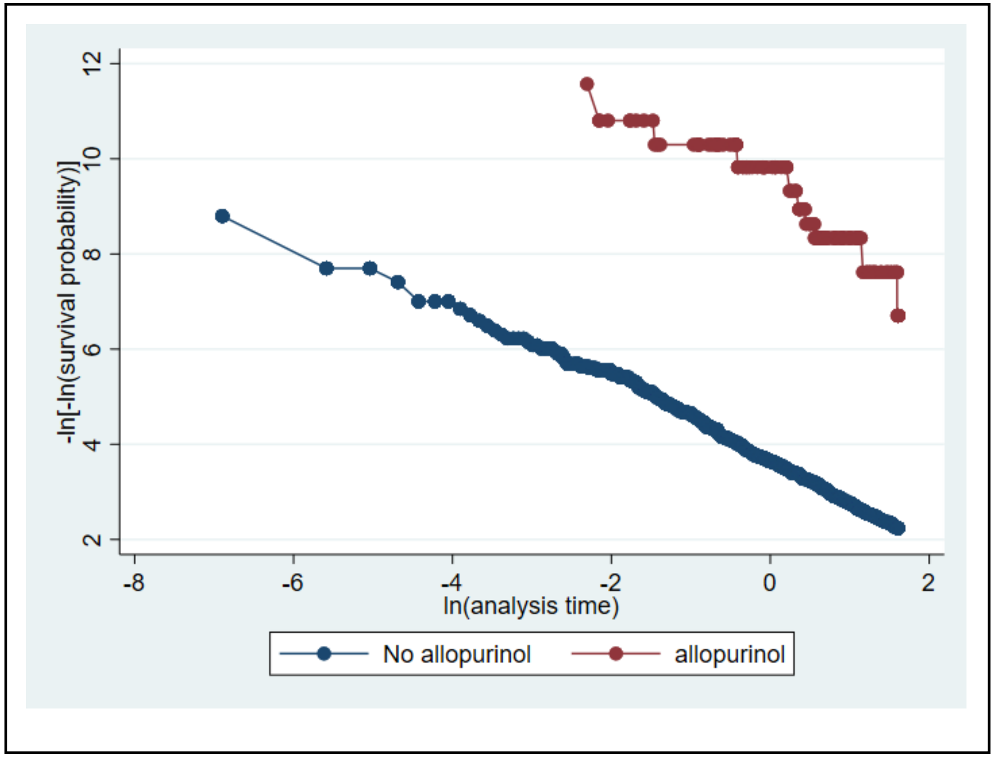

J. ACE Inhibitors

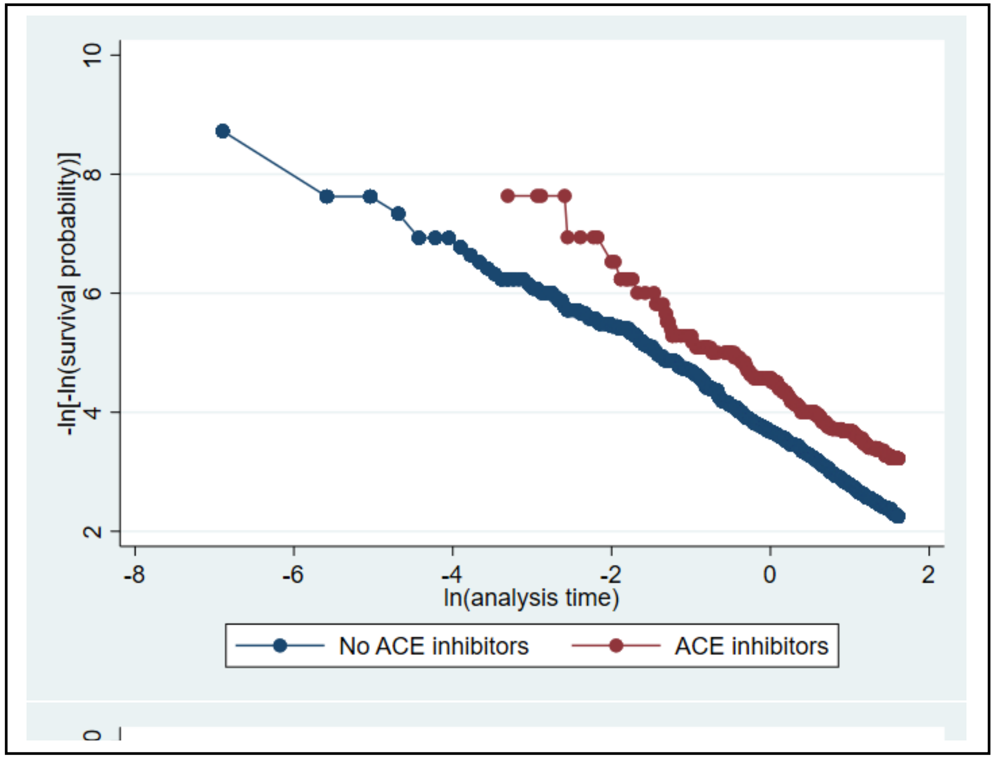

K. Age (years).

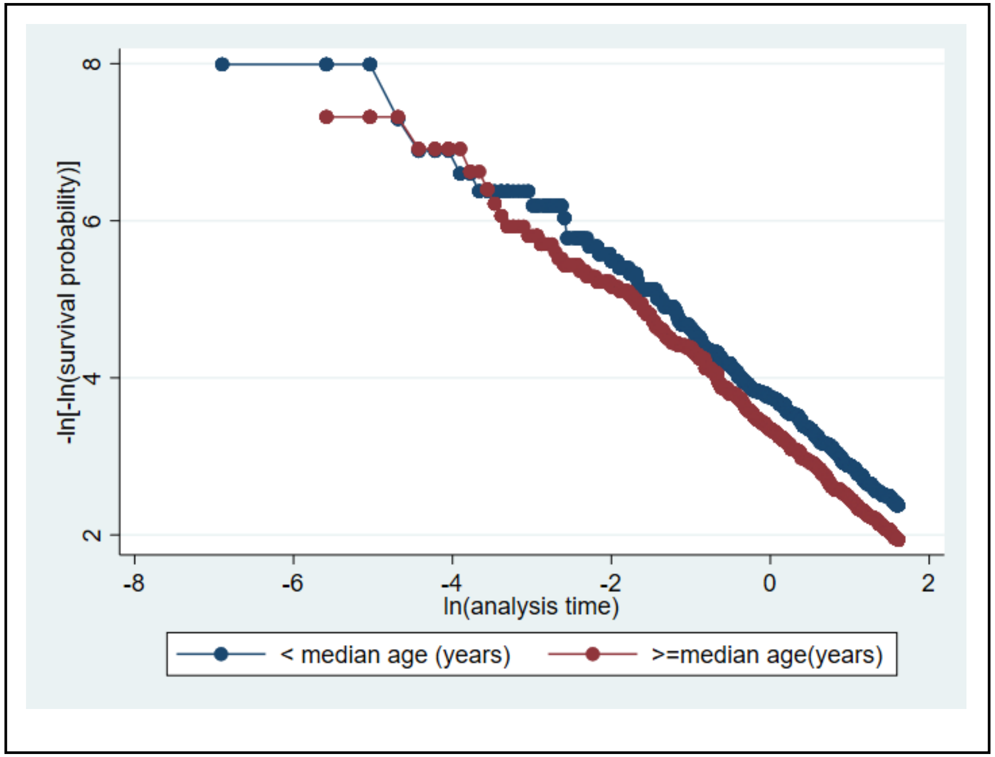

L. Mercaptopurine equivalent dose, (mg/day)

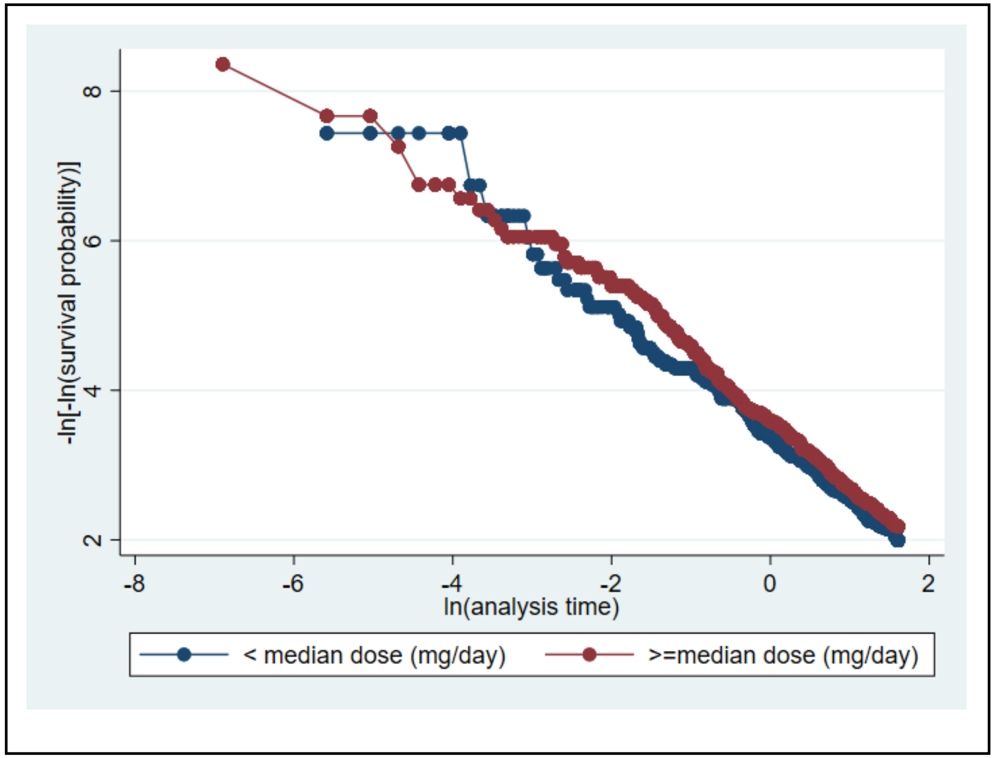

M: Body mass index (kg/m2)

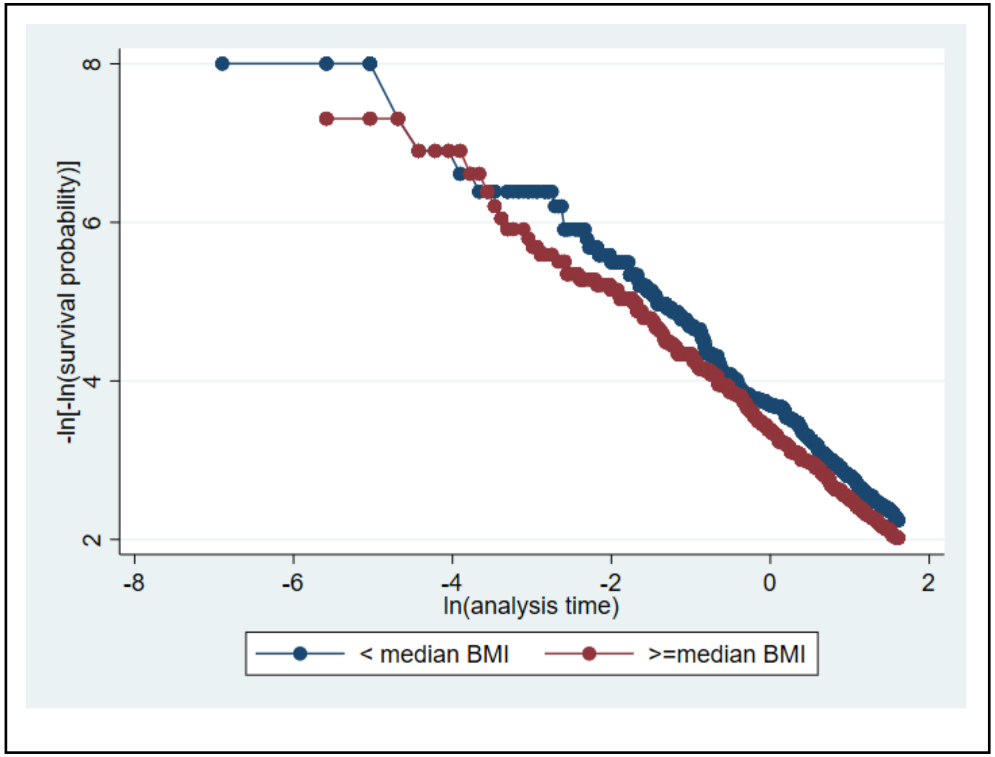

N: At least mild cytopenia or liver enzyme elevation

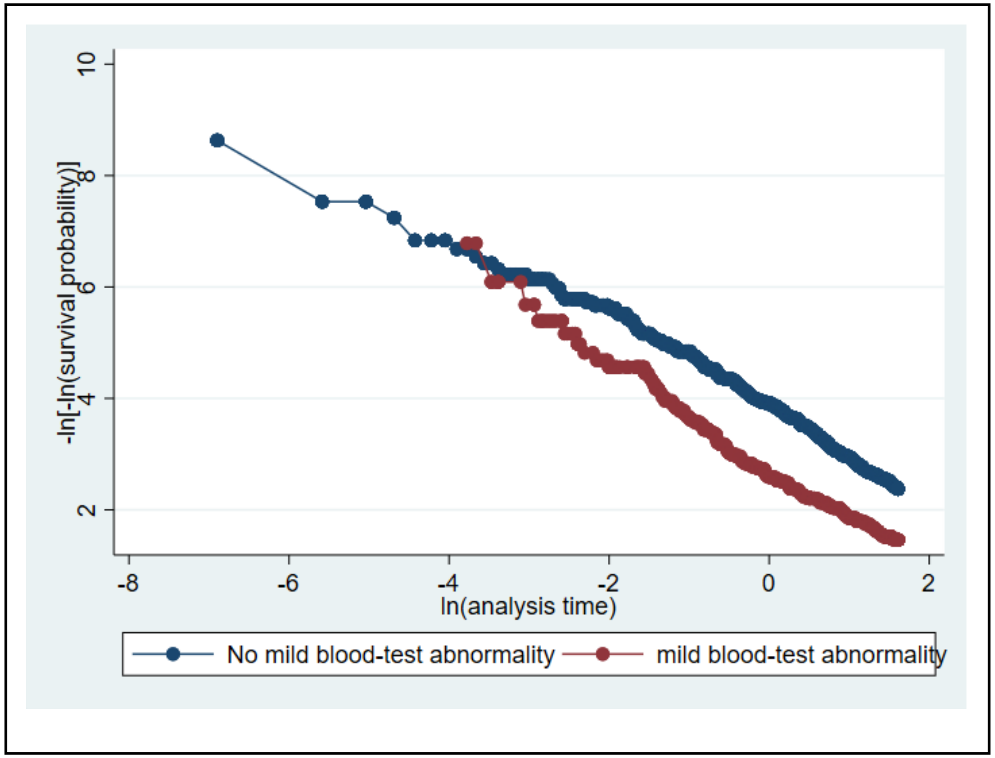

Figure S2: Distribution of predicted risk in the model derivation cohort at 5 years

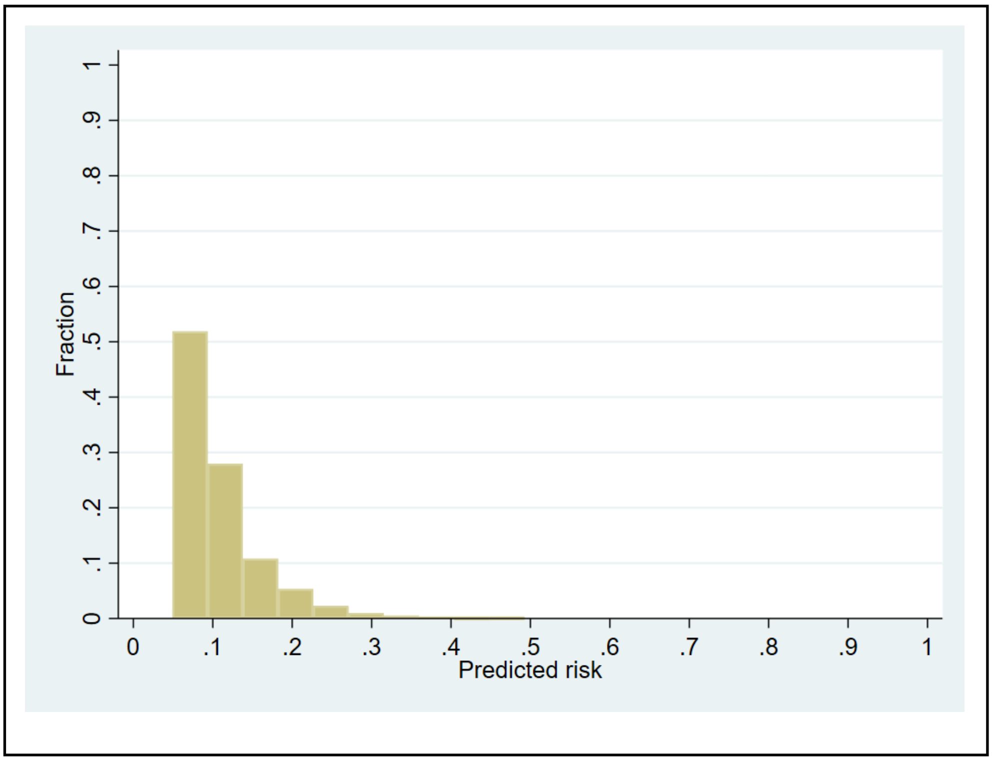

Figure S3: Calibration of a prognostic model for thiopurine discontinuation with abnormal monitoring blood-test results at 5-years in the development cohort

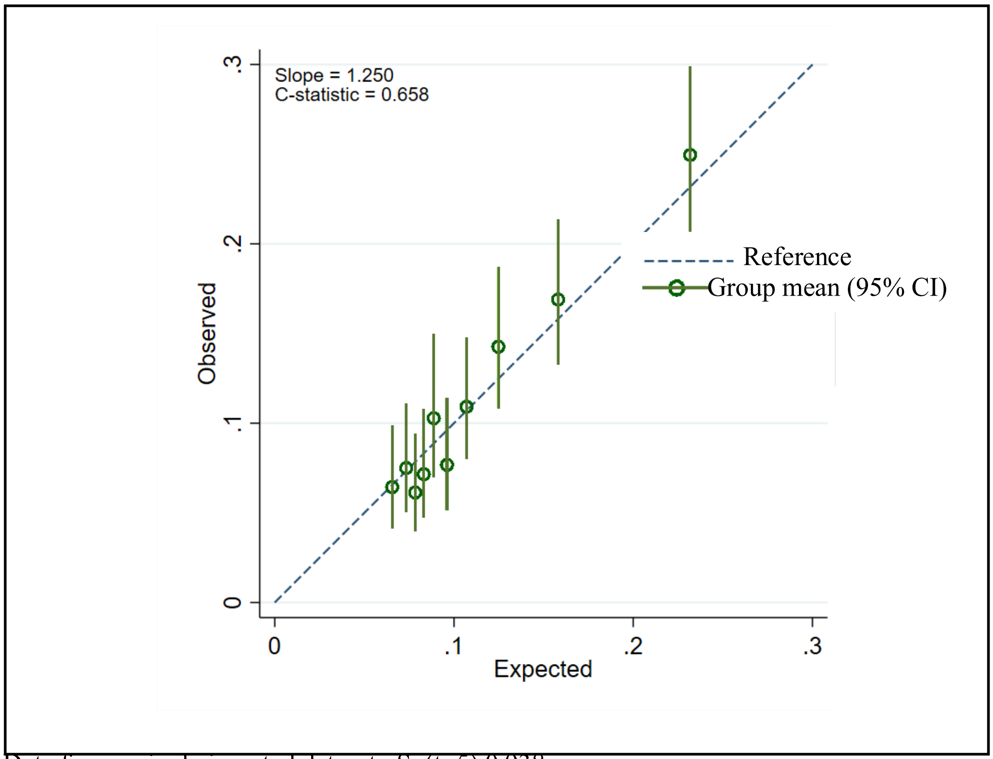

Data from a single imputed dataset; So(t=5) 0.938

Figure S4: Distribution of predicted risk in the model validation cohort at 5 years

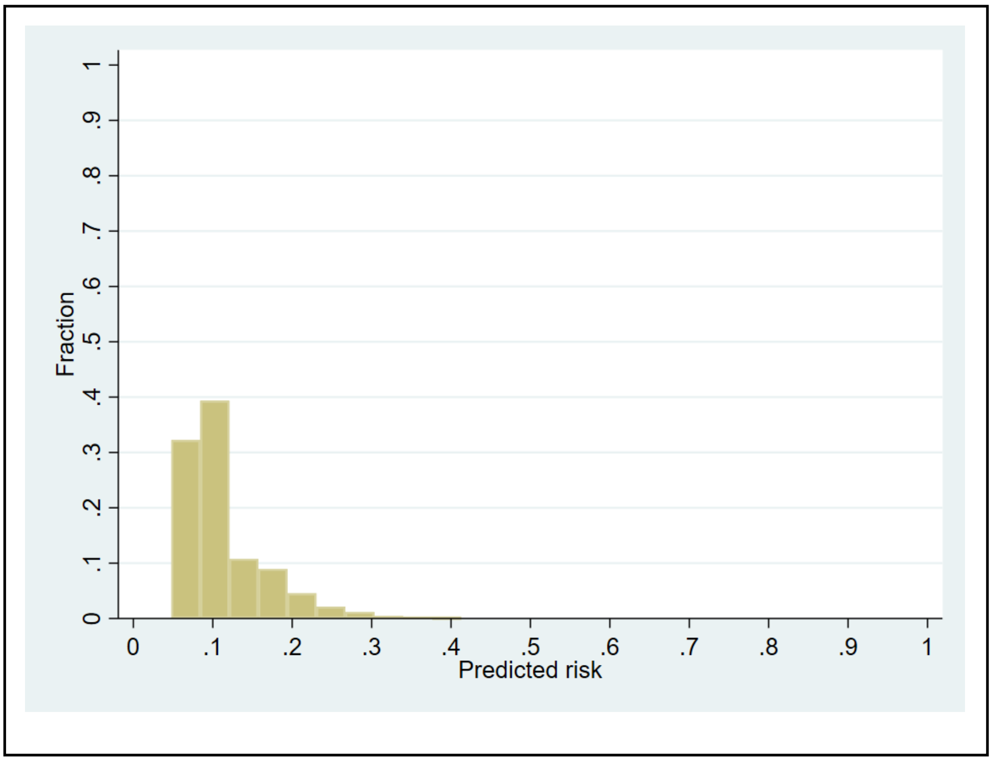

Data from a single imputed dataset; So(t=5) 0·938

Figure S5: Calibration of a prognostic model for thiopurine discontinuation with abnormal monitoring blood-test results at 5-years in the validation cohort

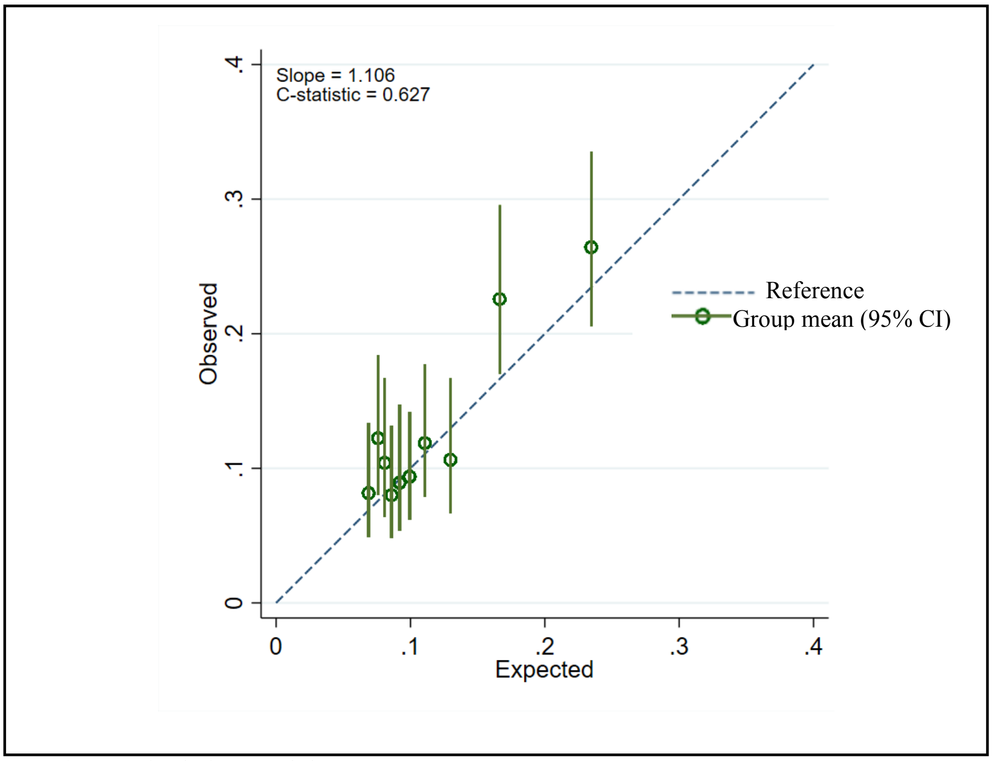

Data from a single imputed dataset; So(t=5) 0·938

Figure S6: Calibration of a prognostic model for thiopurine discontinuation with abnormal monitoring blood-test results at 1 year in the validation cohort

A: Calibration plot

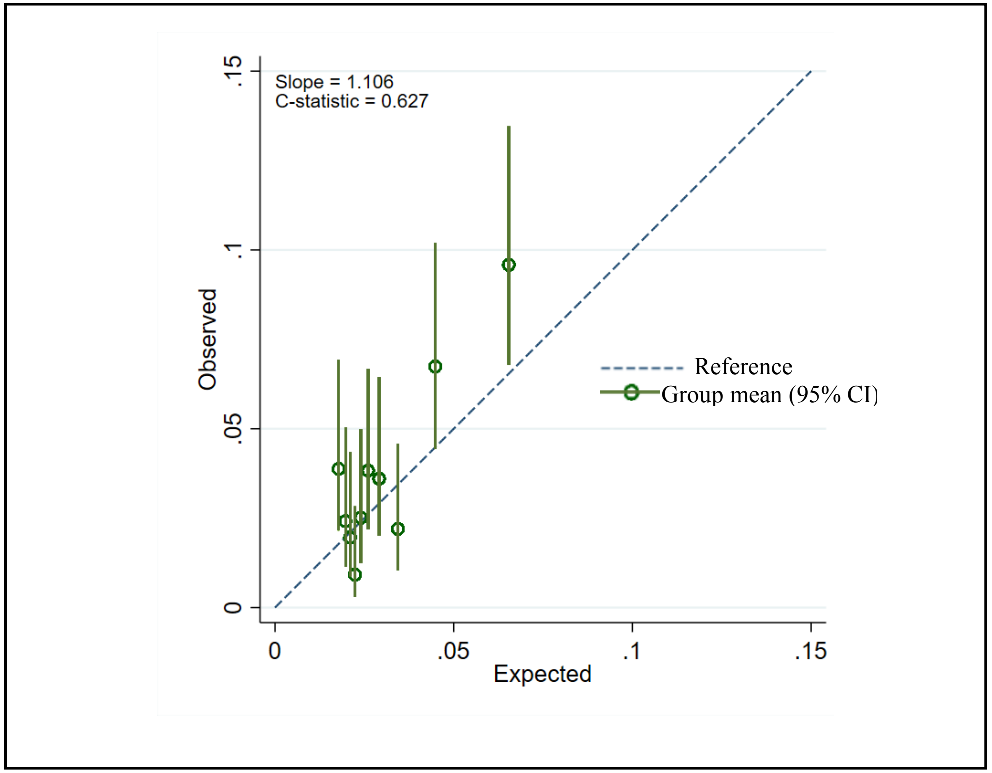

B: Smoothed calibration curve

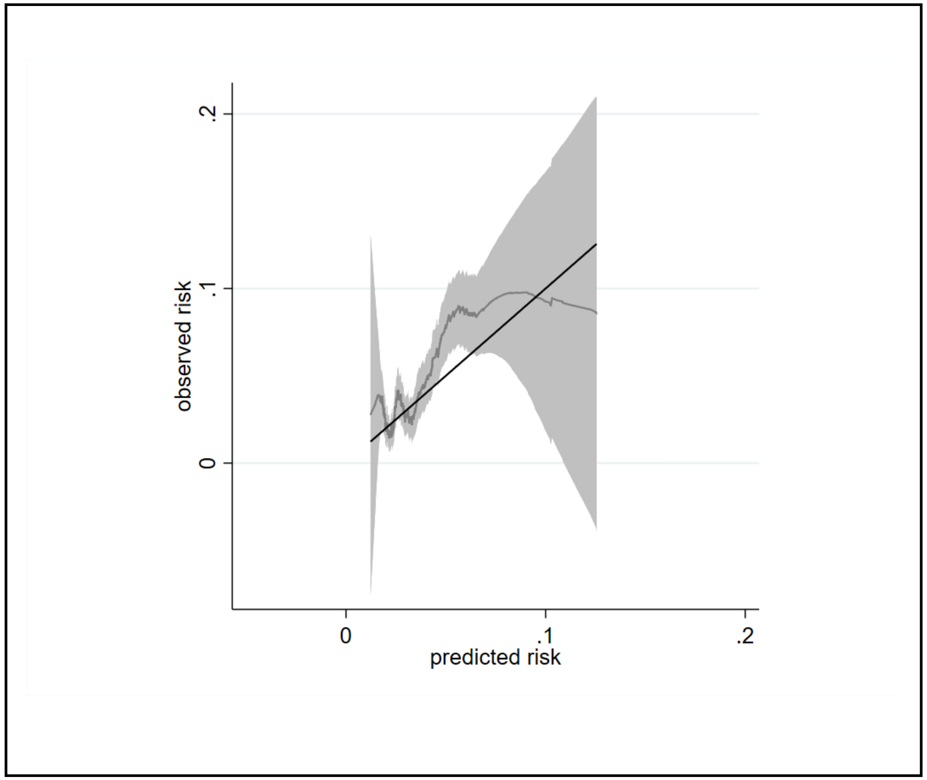

Data from a single imputed dataset was used; $S_0(t_{=1})$ 0·984

Figure S7: Calibration of a prognostic model for thiopurine discontinuation with abnormal monitoring blood-test results at 2 years in the validation cohort

A. Calibration plot

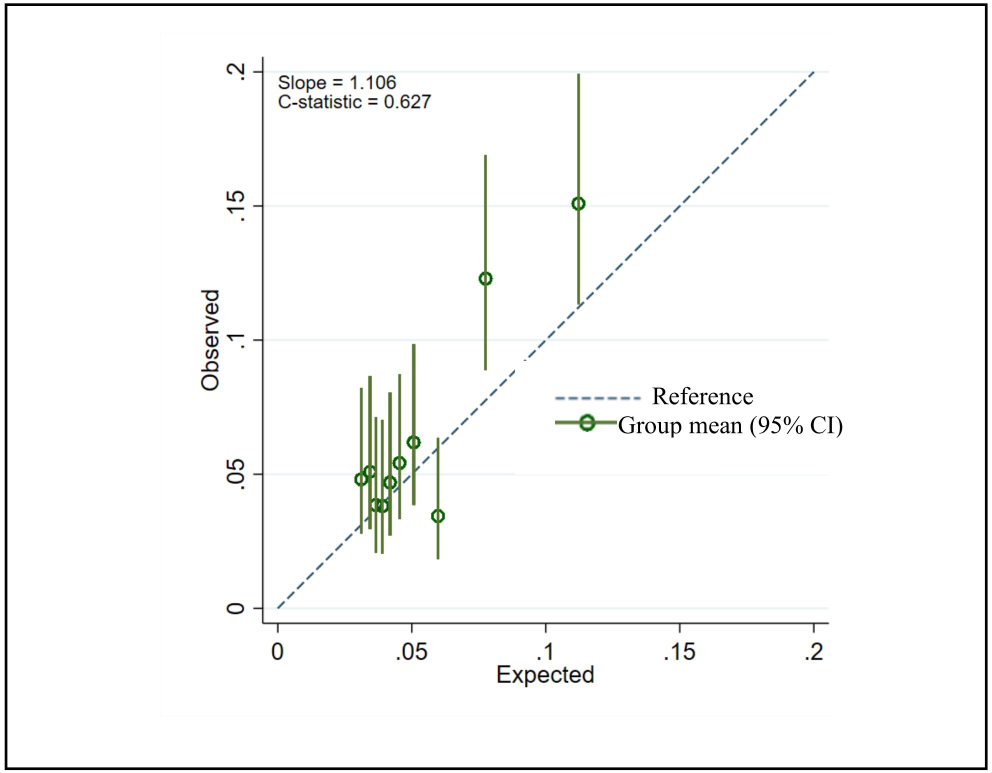

B. Smoothed calibration curve

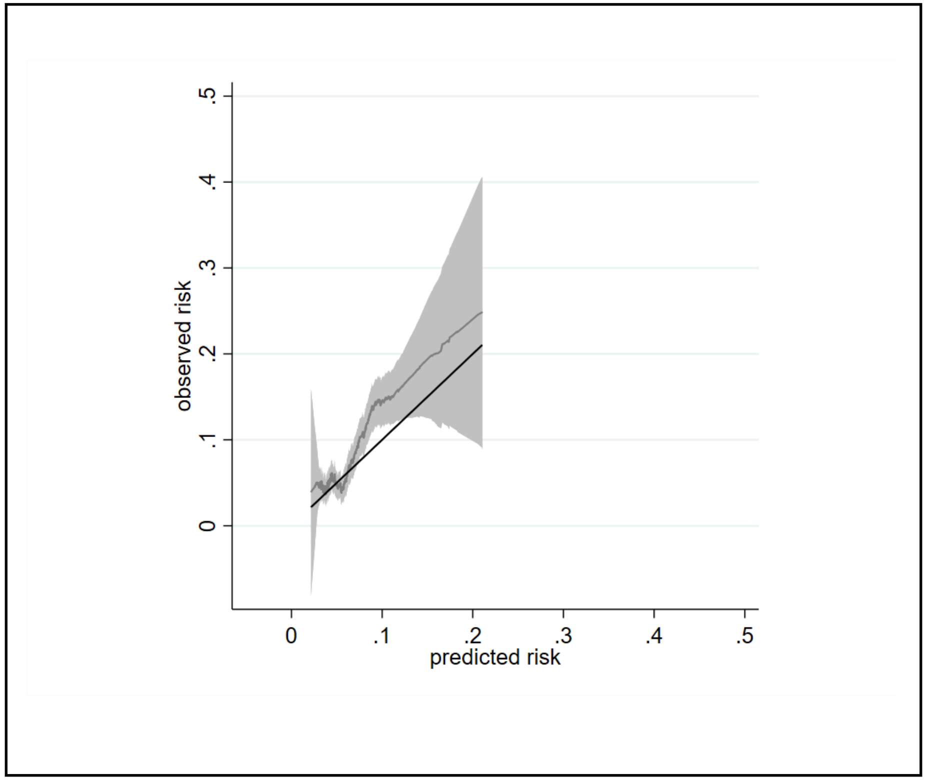

Figure S8: Calibration of a prognostic model for thiopurine discontinuation with abnormal monitoring blood-test results at 3 years in the validation cohort

A. Calibration plot

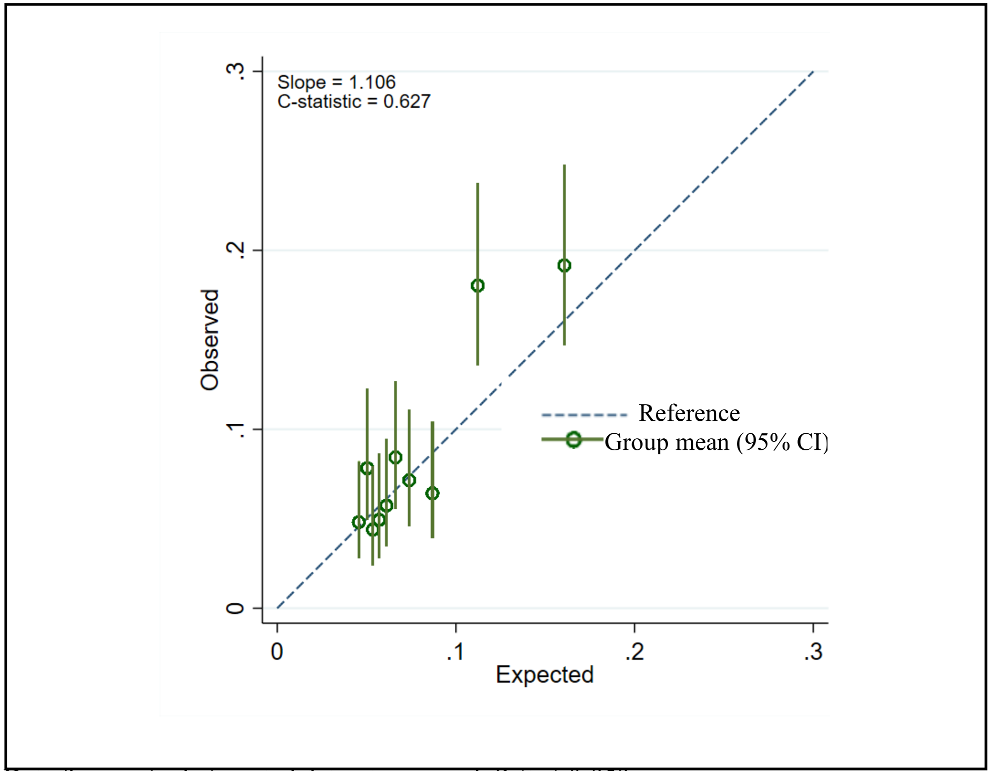

Data from a single imputed dataset was used; $S_o(t_{=3})$ 0·959

B. Smoothed calibration curve

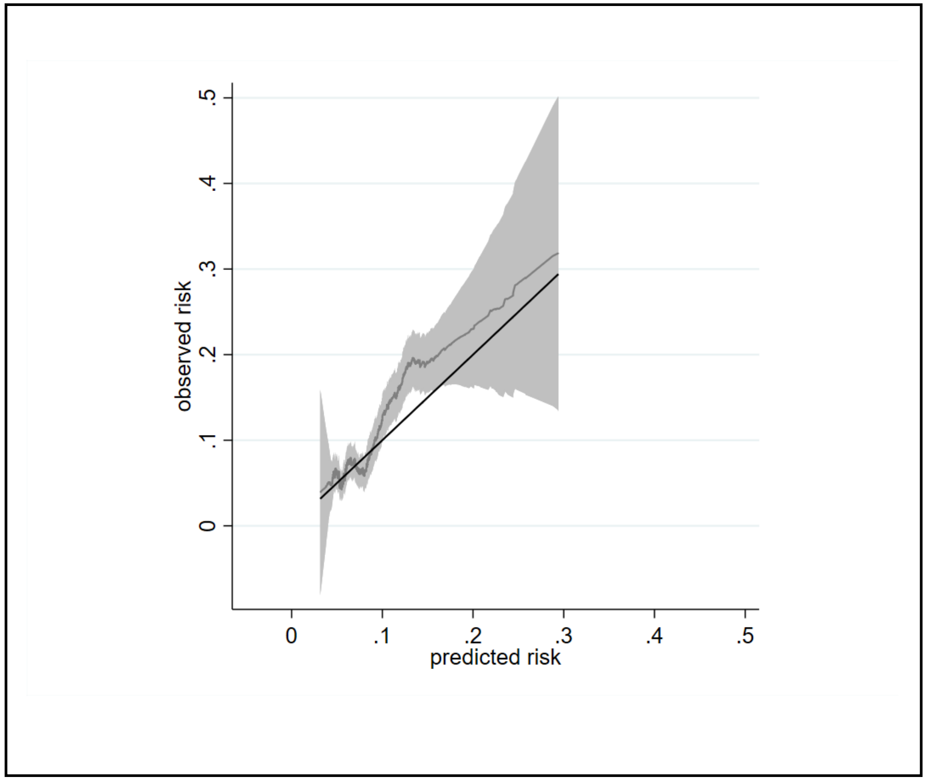

Figure S9: Calibration of a prognostic model for thiopurine discontinuation with abnormal monitoring blood-test results at 4 years in the validation cohort

A. Calibration plot

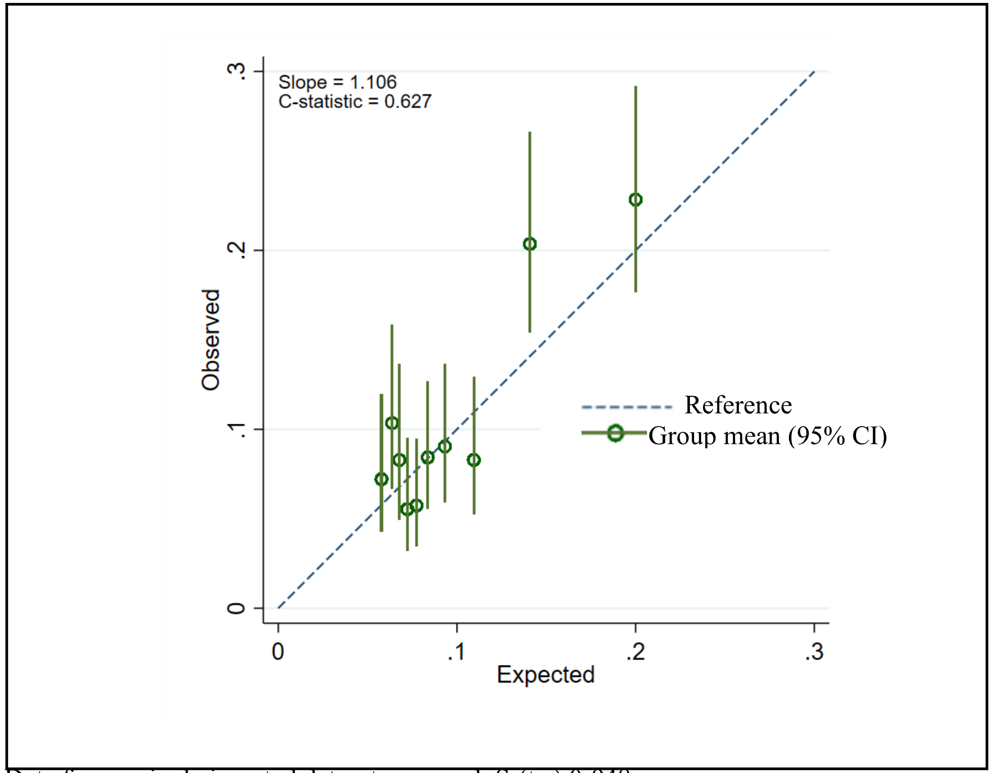

Data from a single imputed dataset was used; $S_0(t_{=4})$ 0·948

B. Smoothed calibration curve

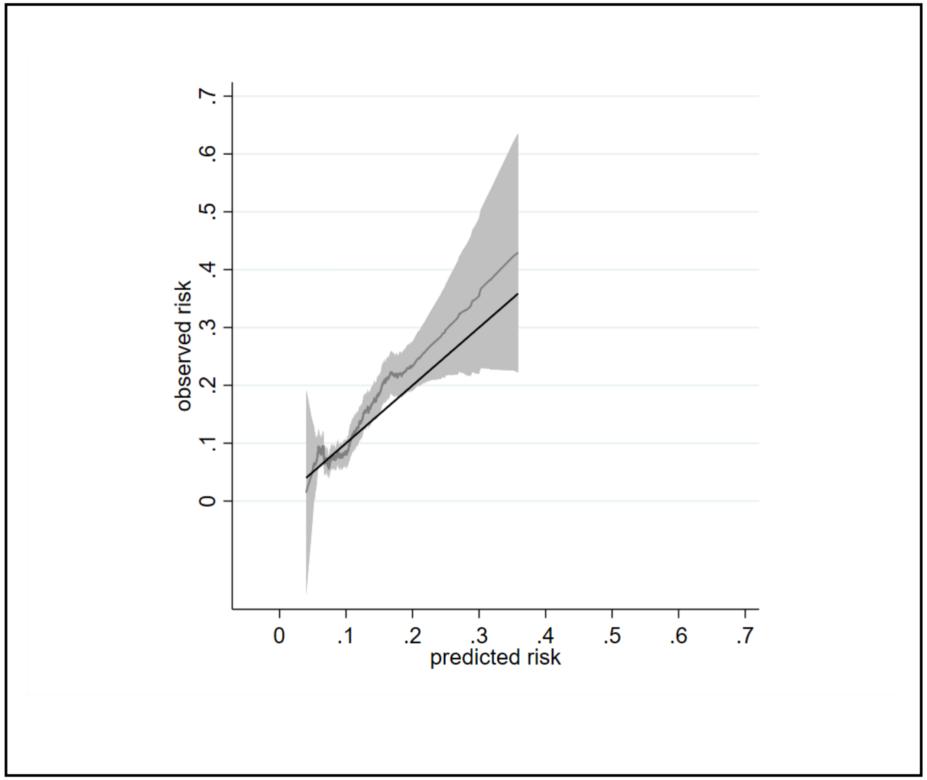

Figure S10: Calibration of a prognostic model for thiopurine discontinuation with abnormal monitoring blood-test results at 5 years in the validation cohort: stratified according to age.

A. <60 years **B. ≥ 60 years**

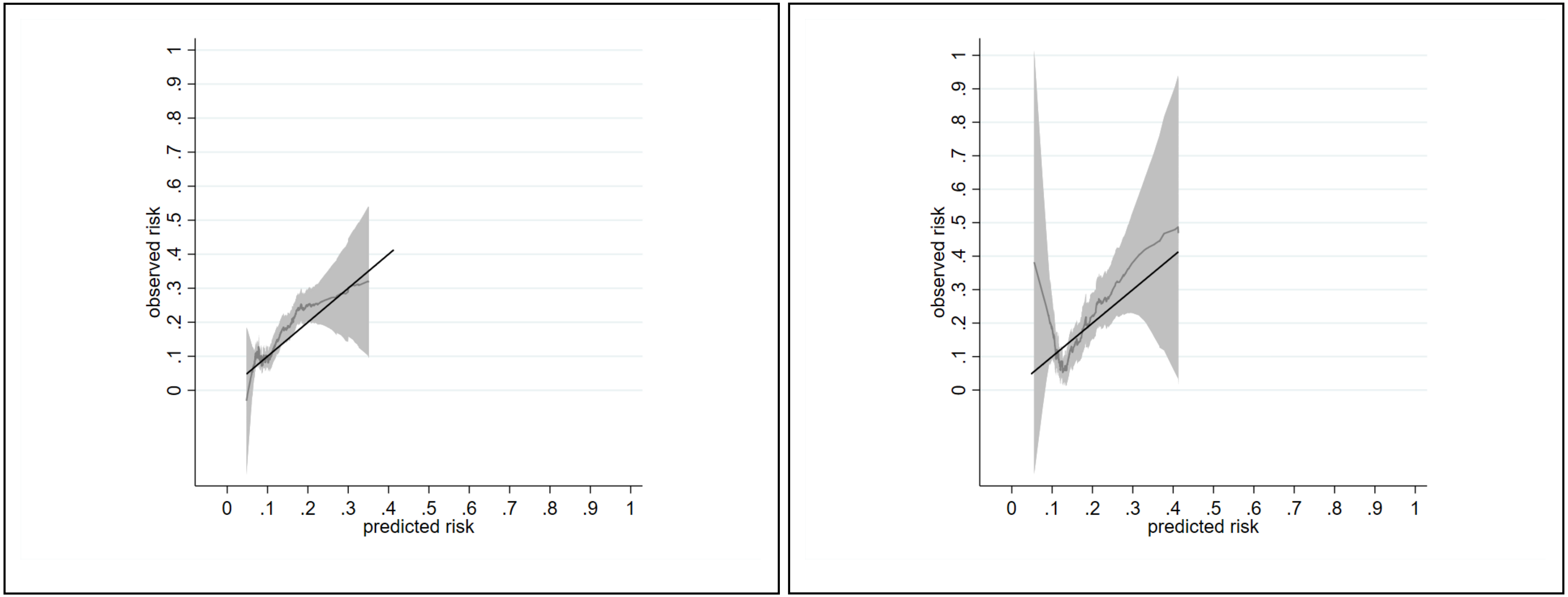

Figure S11: Calibration of a prognostic model for thiopurine discontinuation with abnormal monitoring blood-test results at 5 years in patients who initiated treatment in 2010 or later and IBD: validation cohort.

A: Thiopurine initiated 2010 or later

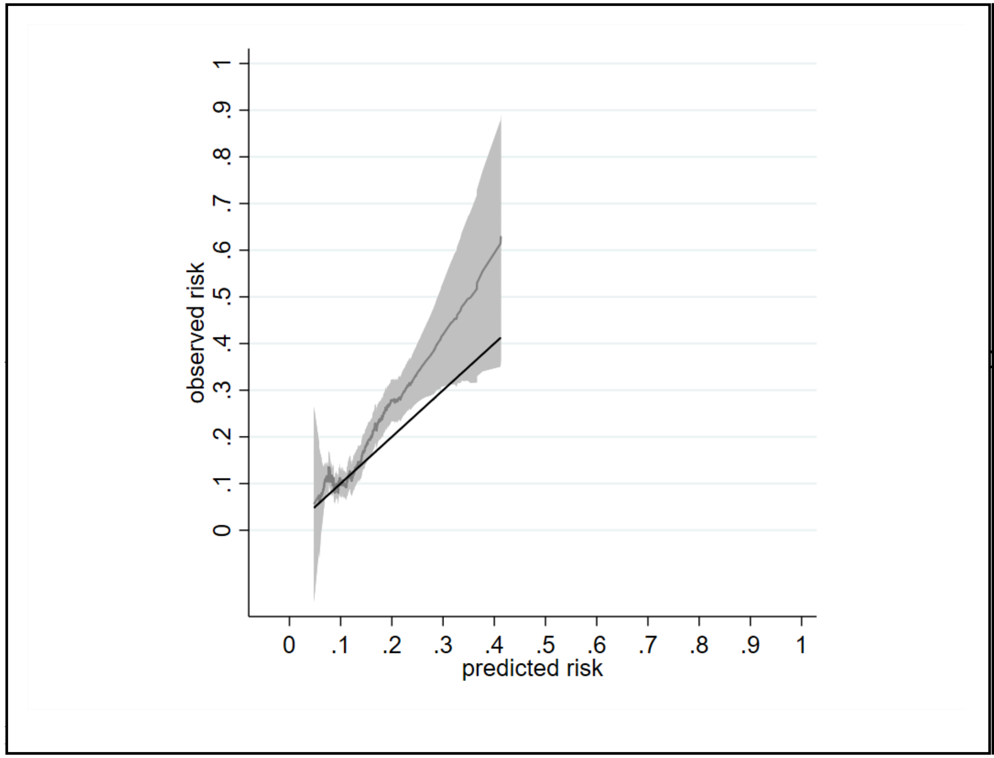

B: Inflammatory Bowel Disease

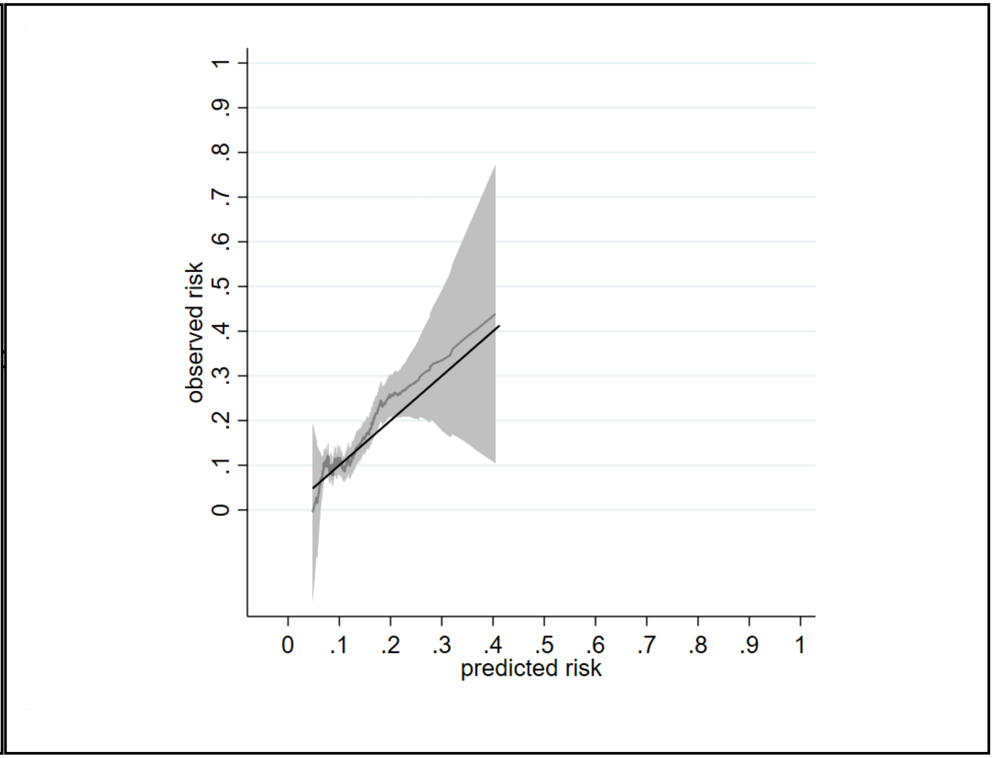

Figure S12: Incremental net monetary benefit results when the risks of serious conditions estimated by clinicians were tripled

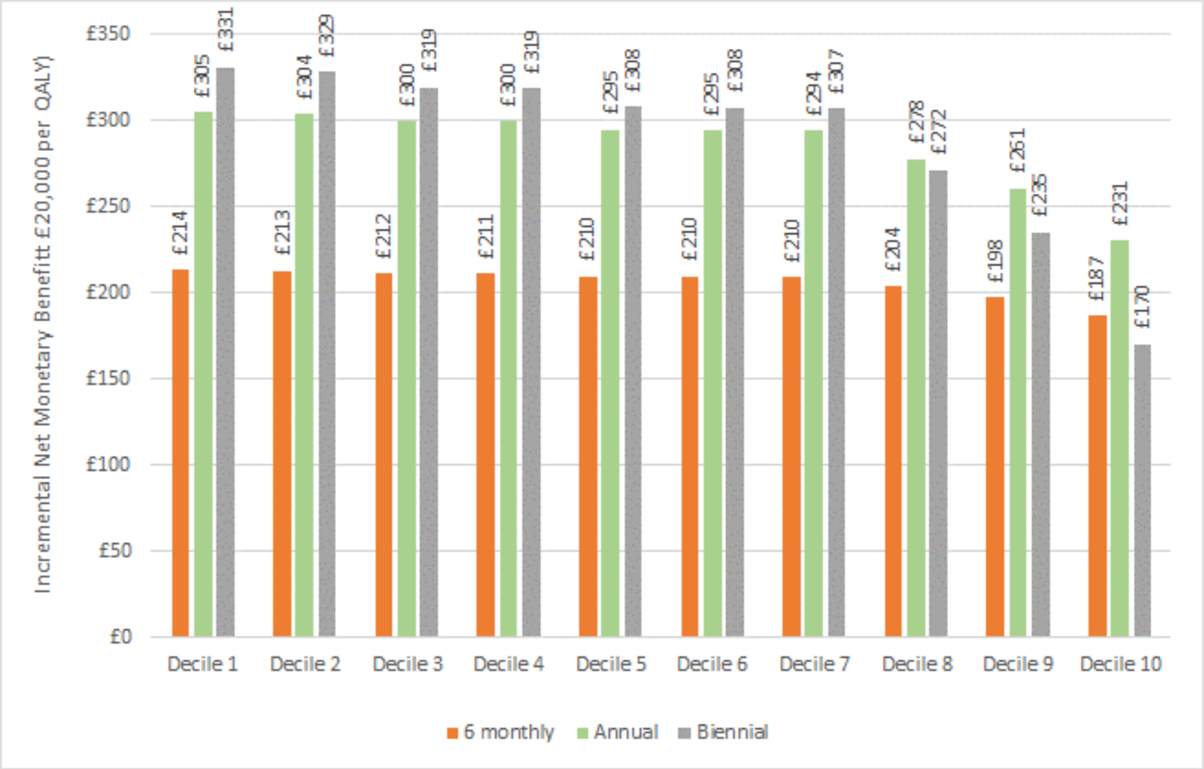

Supplementary references

1. Jones KC, Burns A. Unit costs of health and social care 2021: Personal Social Services Research Unit, Kent, UK, 177 pp. ISBN 978-1-911353-14-0.; 2021.
2. NICE. Single Technology Appraisal Dapagliflozin for treating chronic kidney disease [ID3866] 2021 [Available from: https://www.nice.org.uk/guidance/ta775/documents/committee-papers.
3. Andrade RJ, Lucena MI, Fernández MC, Pelaez G, Pachkoria K, García-Ruiz E, et al. Drug-induced liver injury: an analysis of 461 incidences submitted to the Spanish registry over a 10-year period. Gastroenterology. 2005;129(2):512-21.
4. NICE. Single Technology Appraisal Sofosbuvir-velpatasvir-voxilaprevir for treating chronic hepatitis C [ID1055] 2017 [Available from: https://www.nice.org.uk/guidance/ta507/documents/committee-papers.
5. Stevenson M, Pandor A, James M-S, Rafia R, Uttley L, Stevens J, et al. Sepsis: the LightCycler SeptiFast Test MGRADE®, SepsiTest™ and IRIDICA BAC BSI assay for rapidly identifying bloodstream bacteria and fungi-a systematic review and economic evaluation. Health Technology Assessment (Winchester, England). 2016;20(46):1-246.
6. NICE. Sodium zirconium cyclosilicate for treating hyperkalaemia Technology appraisal guidance [TA599]. 2022.
7. NICE. Non-alcoholic fatty liver disease (NAFLD): assessment and management 2016 [Available from: https://www.nice.org.uk/guidance/ng49.
8. NICE. MRI-based technologies for assessing non-alcoholic fatty liver disease 2022 [Available from: https://www.nice.org.uk/guidance/indevelopment/gid-dg10045/documents.
9. NHS Blood and Transplant. Blood and Components - Cost per Item. Accessed Dec 2022. [Available from: https://nhsbtdbe.blob.core.windows.net/umbraco-assets-corp/26343/price_list_bc_nhs_cost-per-item_2022-23_nocic.pdf.
10. An R, Wang PP. Length of stay, hospitalization cost, and in-hospital mortality in US adult inpatients with immune thrombocytopenic purpura, 2006–2012. Vascular health and risk management. 2017;13:15.
11. NICE. Axicabtagene ciloleucel for treating relapsed or refractory diffuse large B-cell lymphoma after 1 systemic therapy [ID1684] 2023 [Available from: https://www.nice.org.uk/guidance/indevelopment/gid-ta10580/documents.
